# Omnidirectional compliance on cross-linked actuator coordination enables simultaneous multi-functions of soft modular robots

**DOI:** 10.1038/s41598-023-39109-2

**Published:** 2023-07-26

**Authors:** Zhonggui Fang, Yige Wu, Yinyin Su, Juan Yi, Sicong Liu, Zheng Wang

**Affiliations:** 1 Shenzhen Key Laboratory of Intelligent Robotics and Flexible Manufacturing Systems, Southern University of Science and Technology, Shenzhen, China; 2 Department of Mechanical and Energy Engineering, Southern University of Science and Technology, Shenzhen, China; 3 Department of Mechanical Engineering, The University of Hong Kong, Central and Western District, Hong Kong, China

**Keywords:** Mechanical engineering, Electrical and electronic engineering

## Abstract

Earthworms have entirely soft bodies mainly composed of circular and longitudinal muscle bundles but can handle the complexity of unstructured environments with exceptional multifunctionality. Soft robots are naturally appropriate for mimicking soft animal structures thanks to their inherent compliance. Here, we explore the new possibility of using this compliance to coordinate the actuation movements of single-type soft actuators for not only high adaptability but the simultaneous multifunctionality of soft robots. A cross-linked actuator coordination mechanism is proposed and explained with a novel conceptual design of a cross-linked network, characterization of modular coordinated kinematics, and a modular control strategy for multiple functions. We model and analyze the motion patterns for these functions, including grabbing, manipulation, and locomotion. This further enables the combination of simultaneous multi-functions with this very simple actuator network structure. In this way, a soft modular robot is developed with demonstrations of a novel continuous-transportation mode, for which multiple objects could be simultaneously transported in unstructured environments with either mobile manipulation or pick-and-place operation. A comprehensive workflow is presented to elaborate the cross-linked actuator coordination concept, analytical modeling, modular control strategy, experimental validation, and multi-functional applications. Our understanding of actuator coordination inspires new soft robotic designs for wider robotic applications.

## Introduction

**Figure 1:**
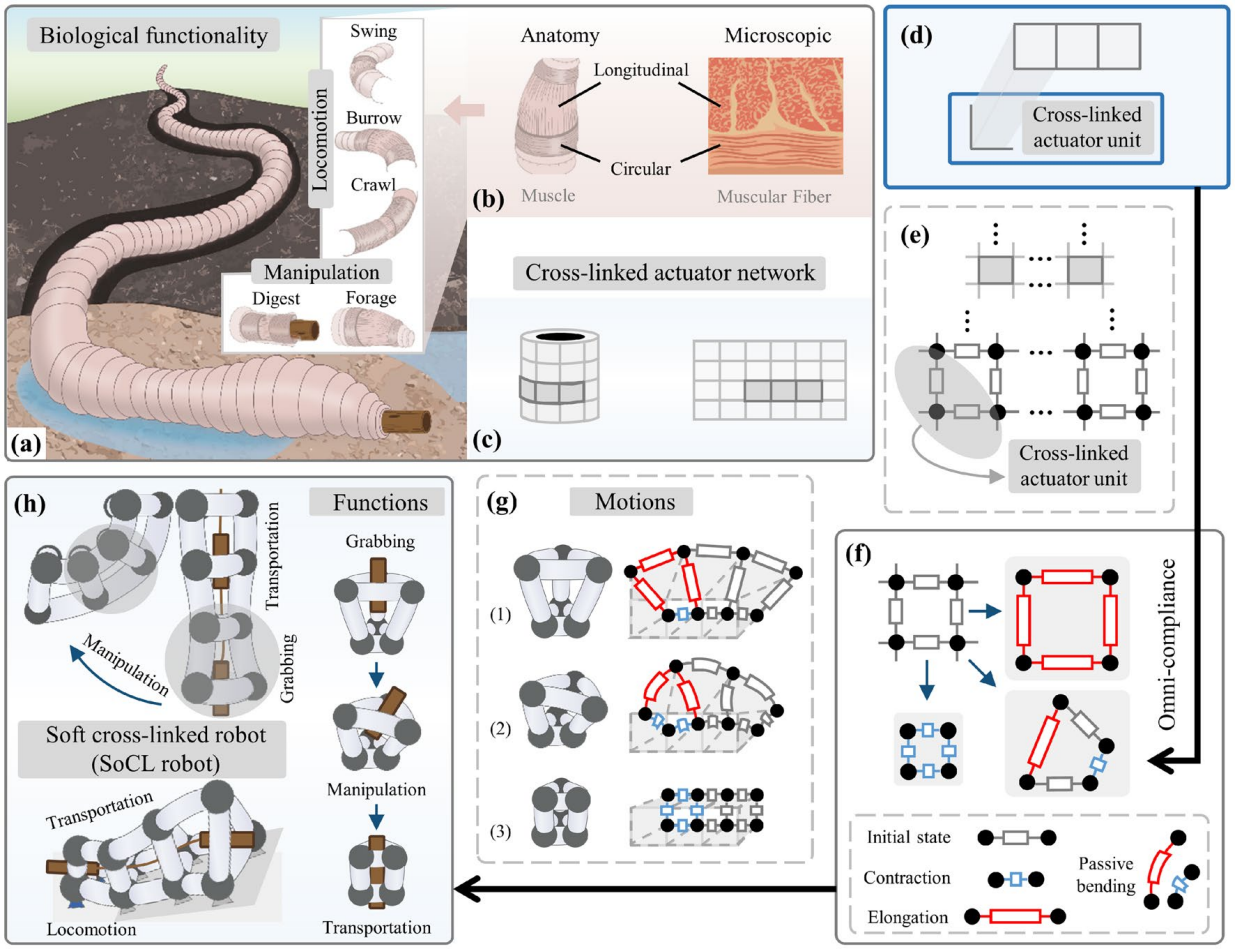
Overview of the proposed concept and approach for simultaneous multi-functions. (**b**) Through modular segments interconnected compliantly by muscular fibers, (**a**) earthworms are capable of multiple behaviors toward manipulation and locomotion. Inspired by this biology insight, (**c**) a cross-linked actuator network was extracted to utilize omni-compliance to achieve simultaneous multi-functions of soft robots. (**d**) This network is composed of the same cross-linked units of single-type actuators (**e**) and has the extensibility of configuration within different scales. (**f**) Thanks to the unit’s omni-compliance, actuators coordinate with adjacent ones to develop multiple deformation forms on the closed ring in two-dimension. (**g**) It is modularly extended to spatial configuration and promotes multiple motion patterns. (**h**) This motion diversity achieves diverse functional operations with the surroundings and objects, facilitating the soft cross-linked (SoCL) robot by stacking segments for simultaneous multi-functions with grabbing, locomotion, manipulation, and transportation.

In nature, earthworms exhibit extraordinary functionality to deal with the complexity of the real world^1–4^. When foraging, the earthworm flexes its body to adapt to the environment and grab food. When burrowing and feeding, the earthworm can move the body and swallow the food simultaneously. They have entirely soft bodies integrated with multiple segments. Each segment is composed of soft muscles with mainly two different sets of arrangement in multiple directions, i.e. longitudinal and circular muscles, as in Fig. 1a,b^4–8^. Such crossed muscle fibers allow a diversity of movements and shape changes. For instance, contraction of the longitudinal muscle fibers causes variation in body length. Simultaneous variation of longitudinal and circular muscle fibers causes complex changes in body shape. As a result, multi-functions could be achieved with specific sequences of these movements and coordination. This relatively simple, functional, and intelligent biological system demonstrates the potential capabilities of a robotic system, thus spurring many robotic researches^9–18^. Among them, soft robots are inherently appropriate for mimicking the soft structures in natural creatures thanks to their inherent compliance, showing a promising perspective in wide robotic applications^10–17^.

The compliance of soft robots is rooted in flexible materials under a wide variety of actuation methods, such as electroactive polymer^19,20^, pneumatically-driven actuator^21–23^, and thermal shape memory alloy^24,25^. Among them, pneumatically-driven soft actuators are superior in biological-muscle-like performance of continuous and compliant movements, thus being extensively investigated in the past decades^26–34^. These remarkable efforts pave the way for the pioneering exploration of functional integration of soft robots. The possibility of combining multiple functions has been explored by integrating several soft actuators in series after each other^35–38^. Although this approach endows the soft robot with not only high continuous and dexterous movements but also high adaptability to unstructured environments by taking advantage of passive compliance, the independent movement of each actuator also results in limited motion controllability and functional extensibility. An interesting variation on the parallel arrangement of soft actuators has been explored and shown higher controllable degrees of freedom by utilizing the compliance among soft actuators with movement coordination^39–43^. More recently, higher functional integration of soft robotic systems has been pioneeringly explored by studying the diversity of morphology with the rigid-soft hybrid structures^44–46^. The combination of the rigid skeleton/joint and soft structure brings versatile movements and high adaptability, while also leading to the local mismatch of performance. Although current research on soft robotics has demonstrated that the inherent compliance endows soft robots with superior capabilities to adapt the unstructured environments, the soft robotic functionality still hardly compares to that of creatures in nature. Therefore, a fundamental understanding of biological morphology and functionality is valuable for new concepts in robotic design and wider applications.

In this work, we explore the new possibility of using this compliance to coordinate the actuation movements of soft actuators for the simultaneous multifunctionality of soft robots. Here, a cross-linked actuator coordination mechanism is introduced to facilitate a novel design of a cross-linked actuator network (Fig. 1c) that utilizes a set of identical soft actuators (Fig. 1d) to be physically interconnected in circular and longitudinal directions (Fig. 1e), completing the construction from a cross-linked element to a two-dimensional (2D) ring (Fig. 1f) and a three-dimensional (3D) segment module (Fig. 1g). Capitalizing on the omnidirectional compliance of soft actuators, the 3D module is able to achieve new motion patterns with simultaneously radial-axial movements that combine radial inflation with the conventional axial linear and bending movements: (1) axial elongation and radial contraction, (2) axial bending and radial inflation, and (3) axial and radial simultaneous contraction. These motion patterns facilitate functions of grabbing, manipulation, and locomotion. By assembling several same modules, a soft cross-linked (SoCL) robot is developed to demonstrate the simultaneous multi-functions with a novel continuous-transportation mode, for which multiple objects could be simultaneously transported in unstructured environments with either mobile manipulation or pick-and-place operation (Fig. 1h).

Overall, the main contributions are summarized as follows: (1) Concept of using the omnidirectional compliance of soft actuators to coordinate the actuation movements, enabling the new soft robotic designs by a single-type actuator for biological functionality. (2) A cross-linked actuator coordination mechanism for the facilitation of a cross-linked actuator network that physically interconnects the actuators from different directions, enabling the simultaneous multifunctionality of soft robots. (3) The whole methodology for full customization of soft robotic systems, involving a novel design of a cross-linked network, modeling of coordinated kinematics, a modular actuation strategy, and construction of a soft robotic system.

## Results

### Conceptual analysis, design, and modeling of the cross-linked actuator coordination

**Figure 2:**
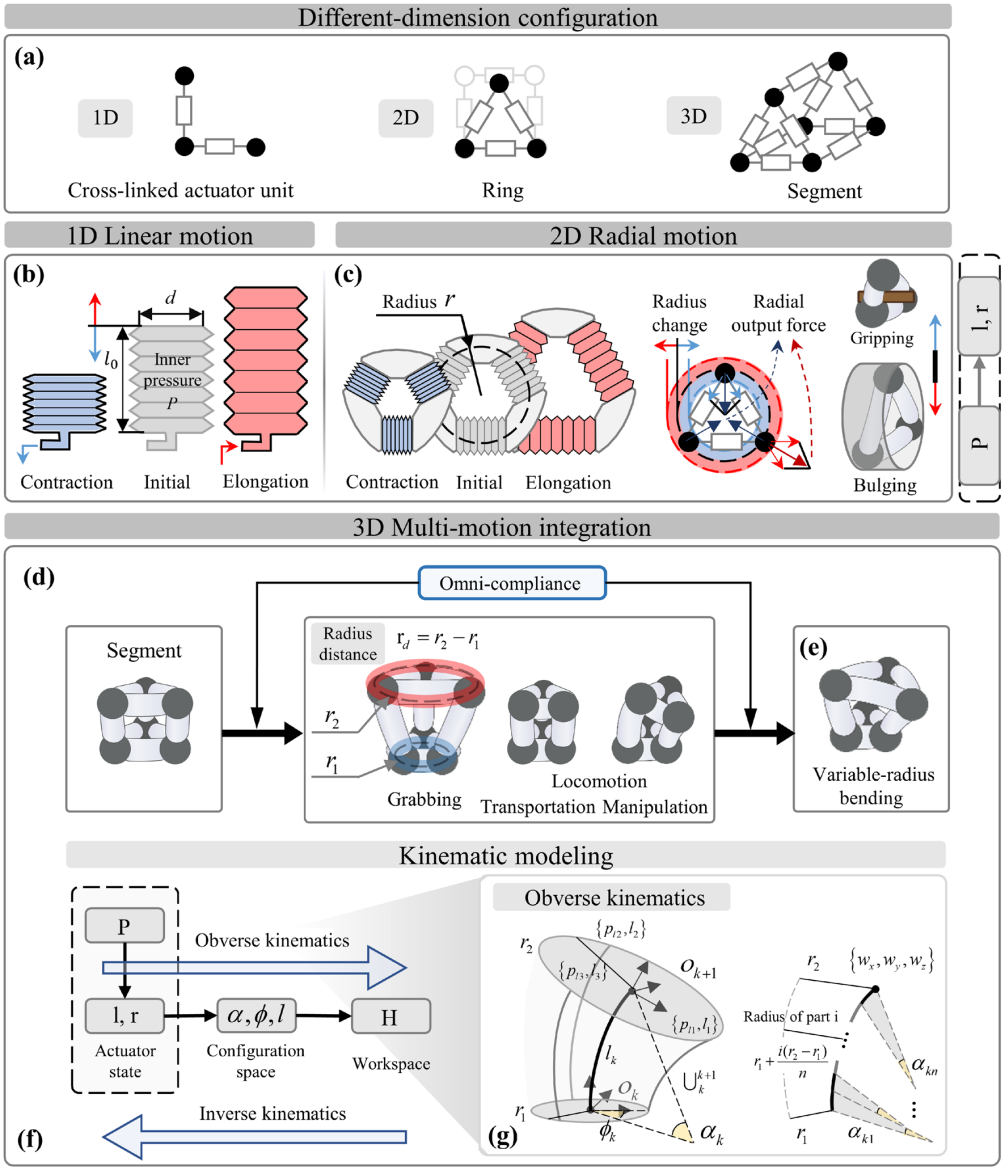
The analysis of configuration and motion characteristics of the approach with modeling. (**a**) The cross-linked network promotes configuration design extended in different dimensions by the same core units, developing the 2D ring and the 3D segment. (**b**) As a case for this approach, the bellows serve as the actuator, enabling the linear motion with contraction and elongation. (**c**) It promotes the ring’s radial motion to develop potential grabbing and bulging functions. With the unit’s omni-compliance, the actuator coordinates each adjacent one to develop multiple motion patterns based on the cross-linked configuration. (**d**) The segment has longitudinal and radial inflation/contraction, with simultaneous bending for integrated movements. (**e**) These motion characteristics promote coupled motions with unique variable-radius bending for potential functional operations. (**f**) The coordinated kinematic mechanism with modeling had been developed, which revealed the relationship between actuator state, configuration space, and workspace (**g**) by piecewise continuous integration within the hypothesis of PCC.

The fundamental unit of the proposed network is composed of two or more intersecting soft actuators at one position. Through physical interconnection, these units enable the expansion of closed configuration for a 2D ring similar to circular muscles (Fig. 2a) and further a 3D segment module by spatial extensibility. To explore coordination forms of the cross-linked actuators using omni-compliance, we employed the simplest configurations involving a ring design by three bellows actuators and a segment consisting by nine bellows actuators (N = 3, with three actuators in parallel and six arranged for two circular rings). These configuration cases guide constructing prototypes to analyze and demonstrate this approach. As a specific example, soft bellows is the unit prototype, which exhibits required active actuation and omni-compliance. It enables bidirectional linear motion by contraction and elongation during changing pressure (Fig. 2b). The ring transforms the linear motion of the bellows into radial motion, developing to interact with the surrounding environment by variable radius and radial output force (Fig. 2c). This promotes potential functionalities such as grabbing and bulging within the configuration, due to its expansion of motion form and omni-compliance to surroundings. Expanding the cross-linked network and leveraging the unit’s omni-compliance, the segment coordinates diverse movements of two rings and parallel bellows, coupled to integrate longitudinal elongation/contraction, radial inflation/contraction, and simultaneous bending (Fig. 2d). The coupled motions of the segment enable unique variable-radius bending (Fig. 2e). These diverse motions bring more interaction patterns with the surroundings, leading to more functionalities. However, these coupled motions from cross-linked actuation with actuator coordination pose investigative challenges.

The state of cross-linked actuators directly influences the execution of coupled motions through omni-compliance for coordination, toward potential separated/simultaneous multi-functions. To analyze the underlying mechanism of actuator coordination, we have developed modular coordinated kinematics with a bidirectional model that depicts the deformation of coupled motions under cross-linked actuation. Specifically, the model primarily focuses on the actuator’s state during actuation, considering the design parameters, as well as the spatial kinematics that establishes the connection between the actuator state, the configuration space, and the workspace (Fig. 2f). Among them, the actuator state model encompasses the motion characteristics (displacement and output force) of both the single actuator and the ring, which are directly influenced by the performance of the selected actuator and the design parameters. Meanwhile, the kinematic model aims to establish the relationship between the cross-linked actuator state and diverse functions’ motion performance and boundaries. It is a generalizable model guidance for customized design and actuation of the cross-linked configuration.

Regarding the actuator state model under the prototype of pneumatic bellows, changes in pressure $\Delta P$ predominantly induce displacement (linear $\Delta l$ of the actuator and radial $\Delta r$ of the ring) and output force (longitudinal $F_{l}$ of the actuator and radial $F_{r}$ of the ring) on prototypes. Consequently, the actuator state model in the actuator (one-dimensional (1D) configuration) was represented through static force analysis as follows:

1 $$\Delta l = (\Delta {\text{P}} S - F_{l} )/k_{l} ,$$

where $S$ presents the cross-sectional area of the bellows, and $k_{l}$ is the longitudinal stiffness.

Through geometric analysis and transformation (Supplementary Note 1), Eq. (1) was extended to express the state of the 2D ring as follows:

2 $$\Delta r = \frac{{2\Delta {\text{P}} S\cos (\pi /2 - \pi /N) - F_{r} }}{{4k_{l} \cos^{2} (\pi /2 - \pi /N)}},$$

where $N$ represents the number of actuators in the ring’s configuration design.

Thus, this actuator state model reveals that the configuration’s relative motion is not contingent upon the connectors’ design (the initial state and absolute motion performance are influenced by the connectors’ design) but rather depends on the motion characteristics of the employed actuator.

Additionally, the bidirectional kinematic model had developed to characterize the configuration space and workspace, with key parameters $\alpha$, $\phi$, and $l$ ($\alpha$ represents the curvature angle around the deflection axis, $\phi$ represents the deflection angle around the z-axis, and $l$ denotes the length of the curvature center), based on the previous actuator state. The obverse kinematic model estimates the configuration space and workspace based on the bellows’ length, for planning. Conversely, the inverse kinematic model calculates the desired actuator state by considering the expected configuration state of functions, for control. To achieve this, we assumed that each actuator has a piecewise constant curvature (PCC) ^47^ and divided the 3D segment into n equal parts, each with the same circular radius of adjacent rings (Fig. 2g). By performing successive integrations by each part (Supplementary Note 2), the obverse kinematic model can be solved as follows:

3 $$\begin{gathered} \alpha = \left[ {2\sqrt {l_{1}^{2} + l_{2}^{2} + l_{3}^{2} - l_{1} l_{2} - l_{1} l_{3} - l_{2} l_{3} } /(3r_{2} - 3r_{1} )} \right]\ln \left( {r_{2} /r_{1} } \right) \hfill \\ \phi = \tan^{ - 1} [\sqrt 3 (l_{3} - l_{2} )/(l_{3} + l_{2} - 2l_{1} )] \hfill \\ l = (l_{1} + l_{2} + l_{3} )/3. \hfill \\ \end{gathered}$$

Here, $l_{i} (i = 1,2,...N)$ represents the length of the i-th longitudinal actuator. This kinematic model reveals that the configuration state is uniquely determined under a clear cross-linked actuator state, and the design parameters affect the output by mainly affecting the rings’ radius state.

Besides, we obtained the transition matrix from the configuration space to the workspace, encompassing both the segment and the SoCL robot (Supplementary Note 2), which expanded this model to estimate under modular stacking one. Additionally, the inverse kinematics model was analyzed and developed into an analytical solution equation by substituting the expected configuration space $\left[ {\alpha_{{\text{d}}} ,\phi_{d} ,l_{d} ,r_{1d} ,r_{2d} } \right]$ and targeted position $[w_{{_{x} }} ,w_{y} ,w_{z} ]$ within the feasible region of the segment into the Eq. (3) (Supplementary Note 3). This reveals the estimation of cross-linked actuation for designated coupled motion.

### Separated/coupled motions verification of the unit, ring, and segment

**Figure 3:**
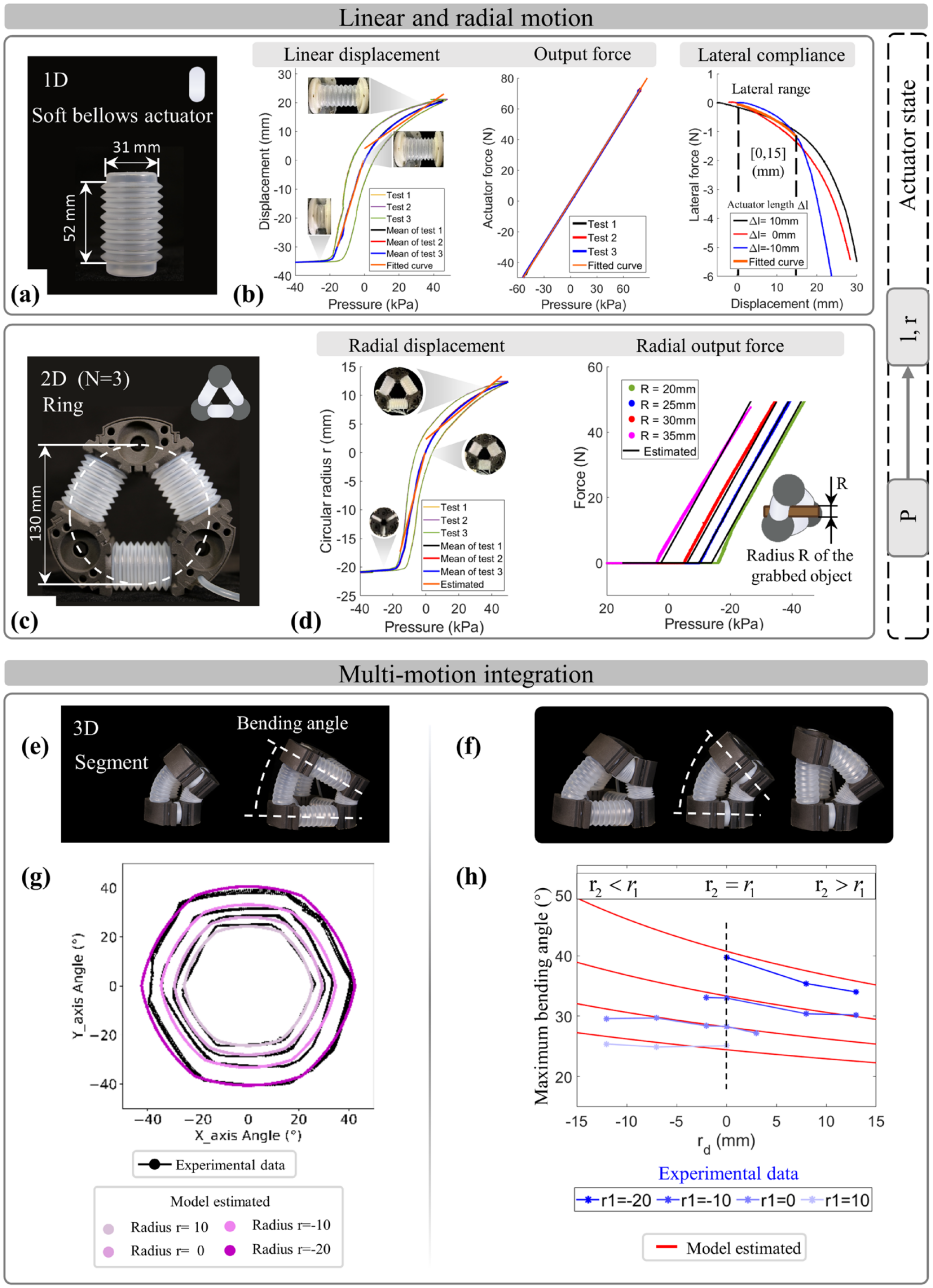
The motion characterization and model validation to different-dimension cross-linked configurations. With the prototypes of (**a**) the bellows, (**c**) ring, and (**e**) segment by the same actuator, (**b**) the motion characteristic of bellows was experimentally tested for the linear displacement and output force and used to calibrate the prototypes’ parameters for a complete model. (**d**) Similar characterizations for the ring’s radial motion were executed to verify the calibrated actuator state model, revealing the performance influence from parameters. (**e**,**f**) The segment as the spatial configuration enabled coupled motion of various-radius bending, which classified and experimentally performed two cases of the segment motion with the constant $r_{d} = 0$ (radius distance) and variable $r_{d}$. (**g**,**h**) The results exhibited the motion characteristics of prototypes and verified the model’s effectiveness and accuracy.

The motion capabilities and modular coordinated kinematic were experimentally validated using prototypes with the actuator (Fig. 3a), the ring with N = 3 (Fig. 3c), and the segment with N = 3 (Fig. 3e) that are all constructed using the bellows, based on the bidirectional model. The following results, corresponding to the spatial kinematic model, should be addressed: (1) The actuation characteristics of the soft bellows actuator were investigated to reveal the relationships between pneumatic pressure, length, lateral compliance, and force. These findings contribute to the modeling of the ring and segment with calibration. (2) The workspace and grabbing force of the ring were quantified to validate the model. (3) The coordinated workspace resulting from the coordination of multiple movements was quantified to verify the capabilities of the soft robotic system preliminarily.

Through calibration (1) to quantify the motion characteristics of bellows and establish the explicit equation of the actuator state model, experiments were conducted to analyze the performance of bellows’ linear motion in terms of displacement and output force during pneumatic actuation (Fig. 3b). For the displacement experiments, the bottom of the bellows fixed while releasing the top, allowing the bellows to move linearly along the slide rail. Conversely, to measure the output force, both ends of the bellows were fixed to limit displacement. The pressure applied to the bellows was systematically varied throughout the experimental process, and repeated tests were conducted (as described in the methods) (Supplementary Fig. S5a,b). The results (Fig. 3b) (the respective data curves shown in Supplementary Fig. S8) show a piecewise linear curve of displacement to pressure (the slope represents the ratio $S/k_{l}$ of cross-sectional area to longitudinal stiffness). A linear output force curve to pressure is also observed (the curve’s slope represents the cross-sectional area $S$). By fitting the experimental results, the bellows’ $S$ and $k_{l}$ parameters were calibrated, providing the determined coefficients for Eqs. (1) and (2). Among them, the value of $S$ represents the output force capability of bellows, and omni-compliance is the combined influence of longitudinal stiffness $k_{l}$ and lateral stiffness $k_{r}$. To evaluate and quantify the bellows’ compliance, a lateral-pull experiment was conducted (as described in the methods). The results (Fig. 3b) show a constant slope of the curve (the lateral stiffness) within the effective lateral range [− 15, 15] (mm). All the calibrated bellows parameters are documented in Supplementary Table S1.

To further validate the accuracy of the calibrated model described in 2, we conducted two types of experiments involving the radius of radial displacement and grabbing force while varying the applied pressure on the ring (Fig. 3c). Comparing with the estimations obtained from the calibrated Eq. (2), the results (Fig. 3d) exhibit the matching curve that displayed a similar trend of piecewise linear radial displacement with varying pressure. Additionally, the results regarding the grabbing force demonstrate precise model estimations. As expected, the grabbing force initially remained at zero and rapidly increased once the ring made contact with objects by its contraction. Notably, the pressure at the inflection point directly correlates to the object’s radius R. This highlights the superior output-force capabilities of the ring compared to single bellows, as evidenced by the steeper output-force curve slope to pressure.

In short, the actuator-state model offers insights into the motion characteristics of the 1D bellows and the 2D ring. It allows us to estimate longitudinal and radial motion (displacement and output force) by the sensed pressure. Additionally, it highlights the impact of design parameters and actuator characteristics on the configuration output, in which the main factors are the actuator’s cross-sectional area, axial and radial stiffness representing omni-compliance, and the actuator’s number used to construct the ring. These have been verified experimentally, laying the foundation for the analysis and effective execution of coupled multi-motions within the 3D segment.

Within the spatial segment of the cross-linked configuration, the kinematic model encompasses a range of motion patterns by imposing constraints on different parameters, including separated/simultaneous longitudinal and radial motion, along with optional bending. To validate the obverse kinematic model, we performed the unique coupled motions of variable-radius bending with two patterns: (1) same radius both of upper and lower rings (constant $r_{d} = 0$) and changing $r_{1}$ (the radius of the lower ring) (Fig. 3e); (2) variable $r_{d}$ and changing $r_{1}$ (Fig. 3f). These experiments aim to validate the model’s accuracy by testing the maximum range of bending angles under different conditions and comparing them with the model’s estimations. In the case of bending pattern (1), the results (Fig. 3g) reveal a hexagon-shaped range of extreme bending angles that expands as the ring’s radius decreases. To represent the bending ability, we took the average of all data points on a hexagonal curve relative to the origin in each case. For bending pattern (2), the results (Fig. 3h) record represented values for various cases (including the cases of bending pattern one indicated by the black dashed line). It observed that the range of bending angle decreases as the $r_{d}$ increases. This observed trend aligns with the kinematic model’s prediction (red curve) and closely matches the values within various cases, effectively validating the model.

Besides, we derived the simulated variable-radius workspace of the segment and the stacked SoCL robot using a transformation matrix (Supplementary Note 2 and Supplementary Fig. S1c–e). Moreover, we verified the inverse kinematic model by simulating various predefined paths (Supplementary Fig. S1f). The coordinated kinematic with bidirectional model quantifies the coupled motions within coordination with omni-compliance during the cross-linked actuation, covering the motion range and configuration status. It reveals the actuator coordination mechanism that the mutual mapping relationship between the actuator state of cross-linked configuration and the motions’ output. It provides model guidance for customizing SoCL robotic systems by the cross-linked actuator approach and operating diverse potential functions.

### Control strategy of multi-functions for the cross-linked actuator coordination

**Figure 4:**
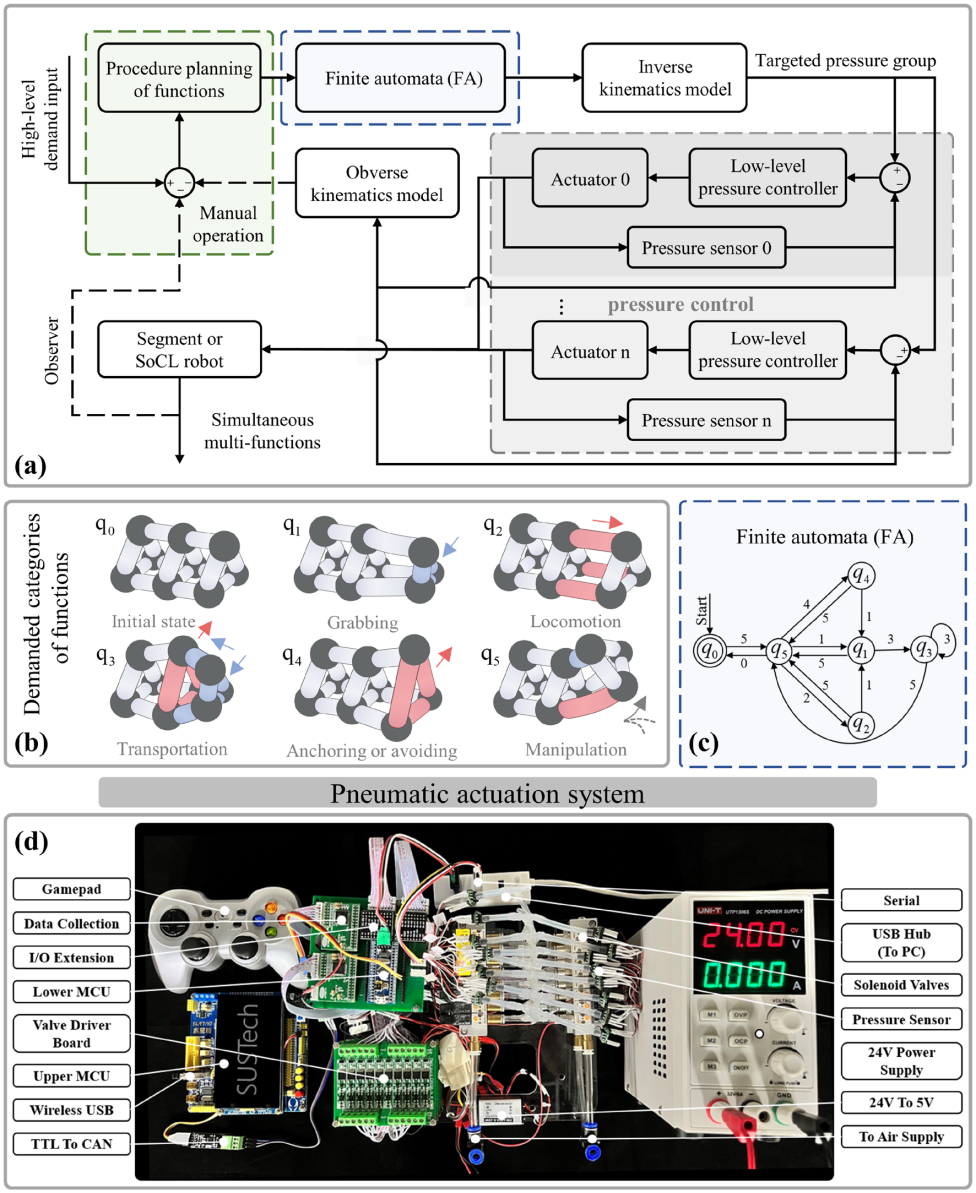
The control strategy based on the analysis of actuator coordination. (**a**) Based on the low-level pressure control, the control strategy manually operates the planning of functions from the input command. It is further input to the finite automata (FA) to transfer to a sequential motion set, which is calculated to the targeted pressure group based on the kinematic model. The closed-loop pressure control regulates the actuators’ pressure of multiple channels to achieve targeted pressures for function execution. Among them, (**b**) the execution basis of simultaneous multi-functions is analyzing actuator coordination state within each separated function. (**c**) These functions were integrated into the modular SoCL robot (or segment) by the finite automata (FA) with a preset route by motion integration. (**d**) A pump-valve system constructed the pneumatic actuation system.

The capability of separated/simultaneous multi-motions offers various interaction forms toward diverse functions, based on the above coordinated kinematic model. For achieving simultaneous multi-functions within the cross-linked configuration, it is crucial to take apart the multi-functions demand in the same time sequence into operable motion integration of the segment, which involves the functional partitioning, estimation of actuator coordination state toward motion of respective functions, and motion combination. Specifically, we artificially divided the functions into six categories (Fig. 4b), in which the ring’s radial motion facilitates grabbing, anchoring, or avoiding, the linear motion and bending of longitudinal actuators enables optional-direction manipulation or locomotion (combined anchoring operation), and the coupled motions of cross-linked actuators enable transportation function (coordinated grabbing and swallowing). As shown in Fig. 4b, each functional submodule has its corresponding motion form for executing during cross-linked actuation (the more comprehensive analysis of diverse functions provided in subsequent chapters), based on the cross-linked actuator coordination mechanism.

The finite automata (FA) is used to integrate multiple motions, including the prioritized selection and combination of motions. Specifically, this compares the motion requirements of each function in sequence following the preset route in Fig. 4c, which can be divided into two situations: (1) when functional motions require actuators within the cross-linked unit are different from the previous, the direct combination of actuator state is not conflicting and achieve the motions’ integration on the segment within the same sequence; (2) when the required actuator state conflicts with each the previous, the function with low priority is set to another modular segment without conflict or waits for the latter sequence with the same motion requirement to combine. Through this operation, the multi-functions demand is reduced in dimension into the comparison and combination between multi-motions into the motion sequences and further mapped to the specific actuator state on the cross-linked configuration, as the guidance for execution instructions. It shows this proposed control strategy’s high simplicity and extensibility due to the simple actuator state set.

For specific experiments and demonstrations on pneumatic prototypes, the input demand is transferred into the actuator state sequences through the procedure of function planning and finite automata (FA), and further converted into the targeted pressure group as input of the low-level pressure control by the inverse kinematic model, as shown in Fig. 4a. It serves mainly as a bridge from the high-level multifunctional demand of task to low-level actuation system, that is a feasible actuation strategy allowed three kinds of inputs: (1) single-step motion of one function; (2) sequential functions of pre-set scripts or trajectories (separated multi-functions); (3) simultaneous multi-functions for tasks.

For the low-level closed-loop pressure control (Fig. 4a), an efficient pneumatic actuation system (Fig. 4d) was constructed using a pump-valve system to regulate multi-channel pressure without mutual interference. For each channel, two solenoid valves (one connected to the positive-pressure source and the other to the negative-pressure source) rapidly adjusted the opening/closing status to maintain the bellows at the preset pressure. With multiple independent channels, precise pressure control was achieved and responded directedly to the demand of case (1). For case (2) and (3), the targeted pressure group were input into the pneumatic controller by temporal order. The subsequent order was executed when the current order was stably attained or the operation’s time exceeded the threshold. By automatically executing all the temporal orders, the pressure group was achieved and further fulfilled potential functions. With high-frequency performance (Supplementary Fig. S2b), the pneumatic actuation system enabled rapid response to sequential inputs, guided by the Lower MCU (Fig. 4d). This capability forms the foundation for executing experiments and demonstrations.

Overall, this analysis reveals the integration mechanism, control strategy, and pneumatic operational process of simultaneous multi-functions within the cross-linked configuration. The effective implementation in the subsequent demonstration confirms the actuation feasibility.

### Separated multi-functions’ analysis and verification of the modular segment

**Figure 5:**
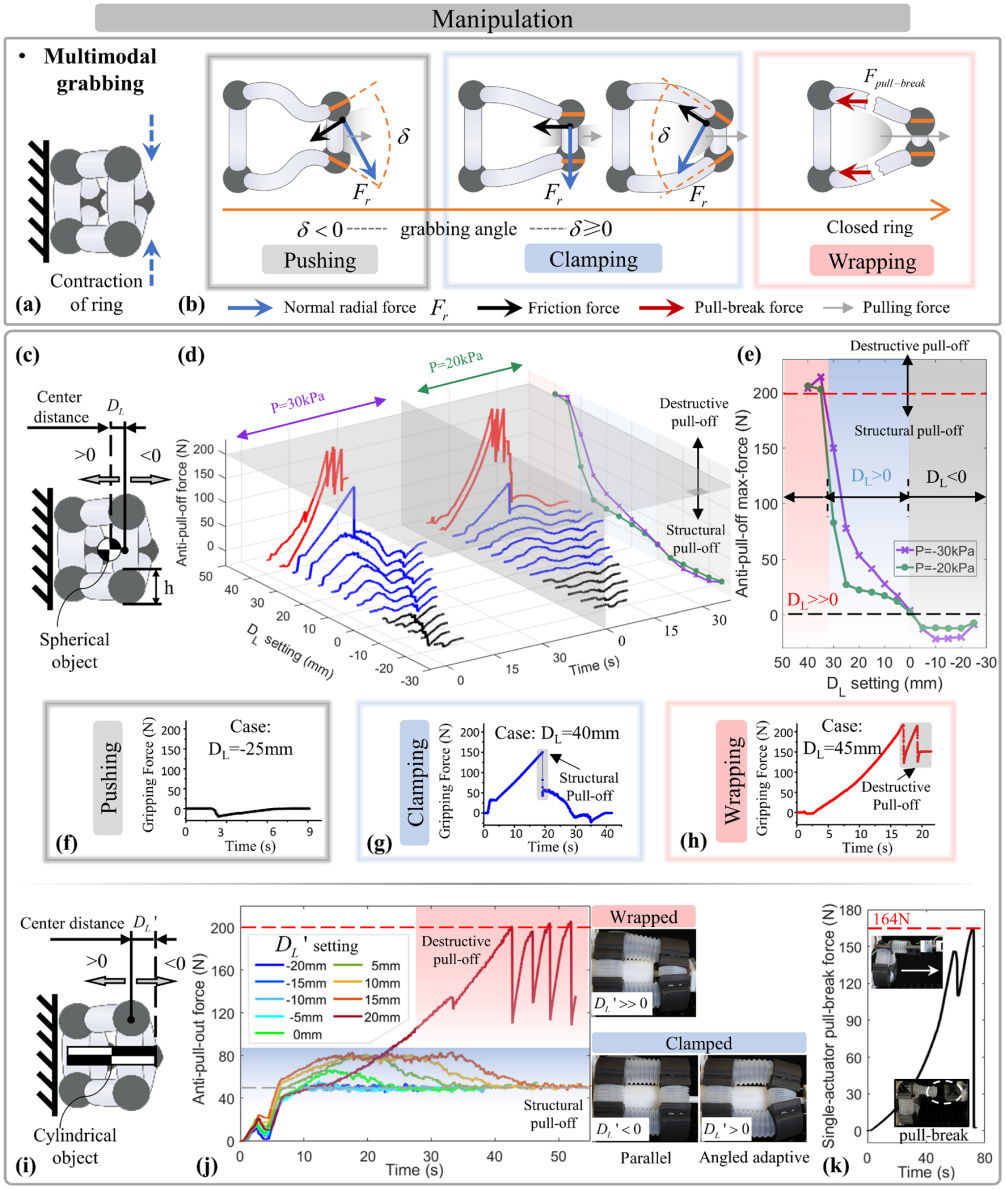
The analysis and experimental verification of multimode grabbing functions. (**a**) Benefit by the ring’s radial actuation and omni-compliant interaction with objects, (**b**) the segment presents adaptive grabbing with a changed grabbing angle δ influenced by the object’s contour, which has different force analysis and divides the three modes of pushing, clamping, and wrapping. For verification (**c**,**i**), we executed the experiment of anti-pull-out force, covering spherical and cylindrical objects with different settings of center distance (change the contacted contour). (**d**,**e**,**j**) It showed increasing grabbing stability (anti-pull-out force) until the novel closure-grabbing pattern with the performance exceeded 200 N (**f**–**h**). The different-modes force processes were matched to the above analysis. (**k**) The pull-break force of the actuator was tested, which influenced the extreme force of the wrapping grabbing.

**Figure 6:**
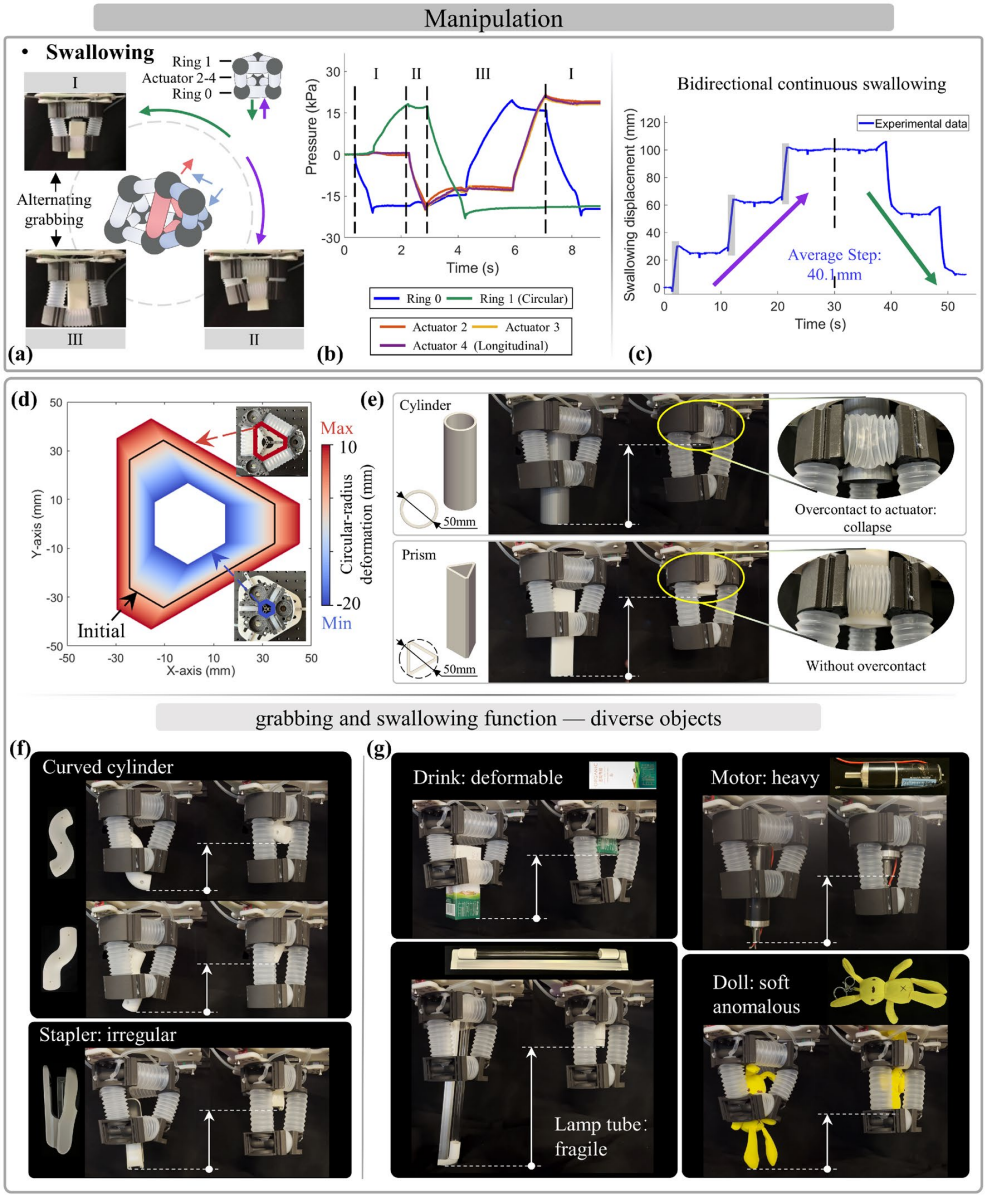
The analysis and operability verification of continuous swallowing function. (**a**) By coordinating the alternating grabbing and longitudinal elongation/contraction sequentially, the segment allows for reversibly and continuously transporting objects within itself. (**b**) Using a predetermined pressure group following the coordinated sequence, it executed a single-loop swallowing, (**c**) the operation of bidirectional continuous swallowing was performed by utilizing multiple loops with stacked pressure. It shows the average step of 40.1 mm. (**d**) The operated objects’ horizontal size was estimated by simulating the ring’s grabbing range. The omni-compliance improves the swallowing adaptability to objects’ shapes, enabling the effective operation to (**e**) over-contacted, curved, irregular, fragile, heavy, or soft objects (**f**–**g**).

The configuration utilizes separated/coupled various motions to achieve diverse manipulation and locomotion functions. In this chapter, to explore the functional principle, performance analysis, influencing factors, applications, and limitations of the functionalities of cross-linked configuration, we analyzed and demonstrated the functionalities of the modular segment separately, with the quantify-performance experiments and validation. Among them, the segment’s functionalities develop manipulation capability of multimodal grabbing (Fig. 5) and unique swallowing (Fig. 6), covering the novel and powerful closure-grabbing pattern and continuous transportation of objects.

Regarding grabbing, the grabbing principle, performance, and the influencing factor during different modes have been analyzed. The actuation of the ring’s radial motion promotes the segment to contact objects for grabbing by the ring’s inner side (Fig. 5a). Affecting by the contacted object’s contour, the grabbing ring generates an adaptive deformation with changed angle $\delta$ due to omni-compliance, unless the ring is completely closed to surround the object. It divides this grabbing process into three modes of pushing, clamping, and wrapping (Fig. 5b). Theoretically, the normal direction of the object contour grabbed is assumed as the direction of normal grabbing force (blue arrow) after adaptation. This is directly determined by the adaptive angle, divided into four situations of anti-pull-out force to resist objects’ escape. As for grabbing inner-convex objects, the angle $\delta < 0$ and the ring’s normal radial force is outward to push the object. While for the parallel or outer-convex contour, the angle $\delta \ge 0$ and the radial force is parallel/inward to keep the object clamped effectively. From pushing to clamping mode, the changed angle $\delta$ continuously increases, and the friction force (black arrow) always plays a positive role in resisting outward pulling force, which leads to the increasing performance of anti-pull-out force (the horizontal combined force of normal grabbing force and friction force) as the angle $\delta$ rises.

Especially within the wrapping mode, the object is completely encysted by a closed ring and cannot escape unless the pulling force forcibly destroys the longitudinal actuators. It reaches the most powerful performance $N*F_{pull - break}$ of grabbing, where $F_{pull - break}$ is the force to pull breaks one longitudinal actuator.

Therefore, the theoretical force limit to resist external pulling force $F_{anti}$ can be estimated through this force analysis as follow:

4 $$F_{anti} = \left\{ {\begin{array}{*{20}c} {F_{r} \sin \frac{\delta }{2} + \left| {\mu F_{r} \cos \frac{\delta }{2}} \right|\begin{array}{*{20}c} {} & {} \\ \end{array} (pushing/clamping)} \\ {N*F_{pull - break} \begin{array}{*{20}c} {\begin{array}{*{20}c} {} & {} \\ \end{array} } & {} & {} \\ \end{array} (wrapping)} \\ \end{array} } \right.$$

wherein $F_{r}$ is the normal radial force from the ring, and $\mu$ presents the frictional coefficient affected by the material and area of the contact surface. The anti-pull-out force $F_{anti}$ represents the grabbing stability, rises by the increase of angle $\delta$, and primarily depends on the grabbed object’s contour when pushing or clamping modes (neglect changes in friction coefficient). Moreover, in the wrapping mode, the anti-pull-out force reaches the most powerful performance that is unrelated to the grabbed object but mainly depends on the actuators’ bond strength. It reveals the functional principle, force performance trend, and influencing factors of grabbing.

For specific operations, the grabbing process operates by two steps of the cross-linked actuation: (1) the ring and longitudinal actuators elongate to surround the targeted object; (2) the ring contract to grab the object and output force. During this process, the adaptation to objects’ contours generates passively due to omni-compliance. Among them, the grabbing stability is essential for effective operation, and its trend is analyzed as above.

To demonstrate the multimodal grabbing function and verify the force analysis of grabbing stability, we implemented the experiment of anti-pull-out force on the prototype (in the methods), covering cases of spherical and cylindrical objects. Note that these are different symbolic cases of verification for comparison. The spherical objects reduce the friction effect by the line contact, while the cylindrical objects amplify the friction effect by surface contact. Experimentally, with the specific object, dissimilar contours at changed positions on the object are used to contact and generate different modes of grabbing for experimental comparison, by adjusting the object’s distance to the grabbing ring.

As for spherical objects, the center distance $D_{L}$ (the distance between the center of the grabbing ring and the center of spherical objects) (Fig. 5c) and the ring’s grabbing pressure were set initially. The results (Fig. 5d) show the measured curves of entire-process force during exogenous pulling until the object was forcibly pulled out. Within diverse settings, all curves can be divided into three categories (different colors) according to the grabbing modes, with separate curve trends during pulling (Fig. 5f–h). The curve’s peak is used as the maximum anti-pull-out force. It was recorded for intuitive comparison, as shown in Fig. 5e. The results (Fig. 5e) present a rapidly rising trend of the anti-pull-out force, as the spherical object’s contour contacted changing by the initial distance increases, in which modes from pushing to clamping and wrapping. This trend matches the theoretical analysis of force. Notably, the wrapping mode shows a powerful performance exceeding 200 N (the extreme value of the motor slipping and not the prototype’s bounds) to resist the external pulling force. Its video of the experimental process (Supplementary Movie S5) shows the pull-out forms in different grabbing modes, revealing the reason for the more robust performance under the wrapping grabbing. In clamping mode, the grabbed object can forcibly open the segment’s ring to escape by the structural pull-off form. While within the wrapping mode, the object cannot escape from the closed ring unless the destructive pull-out form that the longitudinal actuator is compulsively pulled apart. It promotes the novel and powerful closure-grabbing pattern of firmly wrapping objects.

Similar experiments on cylindrical objects have been conducted to exhibit the effectiveness of multimodal grabbing for different-shape objects and to verify the generalizability of force analysis. Similarly, the initial center distance $D_{L} ^{\prime}$ of the cylindrical object (the distance between the center of the grabbing ring and the bottom of cylindrical objects) (Fig. 5i) was adjusted to generate diverse grabbing cases from clamping to wrapping. The pulling force of the experimental process was recorded until the object was pulled out (Fig. 5j). Specifically, as the distance increasing, the contacted contour led to the angle from $\delta = 0$ to $\delta > 0$, and the anti-pull-out force (the extreme value) increased. When the object was pulled out and the grabbing ring was stretched to $\delta = 0$, the measured force in diverse cases of clamping modes returned to the same value (the theoretical friction force). When a tightly closed ring initially wrapped the object, the anti-pull-out force significantly improved, exceeding 200N. To further explore the theoretical maximum force $N*F_{pull - break}$ of prototypes in wrapping mode, we conducted the pull-break experiment of a longitudinal actuator, which showed the pull-break force $F_{pull - break} = 164N$ (Fig. 5k). These trends and phenomena in the results are consistent with the force analysis mentioned earlier.

These experiments, which involved different objects, effectively confirmed the theoretical analysis of grabbing and unveiled the principle of grabbing modes, force interaction, performance estimation, and influencing factors. Overall, the cross-linked configuration promotes the single-type actuators to enable multimodal grabbing, in which the anti-pull-out force is directly related to the adaptive grabbing angle $\delta$ influenced by the grabbed object’s contour. The anti-pull-out performance is enhanced from pushing to clamping modes. It reaches its peak during wrapping mode due to the novel closure-grabbing pattern. Specifically, the prototypes have exhibited grabbing stability (resist being pulled out) of over 200N during the wrapping mode.

As for the swallowing function, this is efficiently implemented through the sequential execution by coordinating alternating grabbing and longitudinal transfer, developing the capability of bidirectional and continuous transportation of objects (Fig. 6a). As an analyzed example, the process of swallowing upward can be divided into three steps of cross-linked actuation: (1) the lower ring contract to grab objects tightly; (2) the longitudinal actuators contract to transfer the object upward; (3) the upper ring grabs the object and then the lower ring elongates to release for handover, then the longitudinal actuators can elongate to prepare the next swallowing process. This process can be carried out in a cyclic/reverse manner for bidirectional and continuous operation. Experimentally, the sequential pressures were regulated to achieve the single-loop swallowing based on the setting steps (Fig. 6b). The prototype demonstrated bidirectional continuous swallowing by integrating multiple loops of pressures, with the average step of 40.1 mm (Fig. 6c) (Supplementary Movie S6).

It is significant to clarify the boundary and limitations of swallowing functional application and the influence factors on its performance to promote the generalizable design by the proposed approach. Theoretically, the influencing factors to swallowing involve the matching degree between the ring’s effective grabbing (deformation) range, the size and shape of objects, and the actuator’s anti-collapse capability. In which the ring’s capacity can be simulated by the above actuator state model of the Eq. (2). Specifically, overlarge or protruding-shaped objects hinder effective grabbing and further swallowing due to the object forcing the ring’s actuator to collapse. The overlarge difference in objects’ radius contacting by the upper and lower ring may cause excessive distortion and collapse of the longitudinal actuator during its contraction. For effective transfer, the objects’ height should exceed the sum of the longitudinal actuator’s minimum length and the connector’s thickness. Above these, the omni-compliance promotes the adaption to objects and the operation’s success, even if a slight collapse of actuators. The handling of these points ensures the swallowing of objects after stable grabbing.

To verify this analysis, we simulated the grabbing range of prototypes, visually displaying the size boundary of objects that can be grabbed to swallow. As shown in Fig. 6d, the extreme boundary exhibits a hexagonal shape (with N = 3), in which three bounder lines of actuators should avoid over-contact to collapse. Benefited from the actuator’s omni-compliance for adaptation, even if the cylinder over contacts actuators and forces it collapses (compared to the prism case with suitable contour), it still being effectively grabbed and swallowed, as shown in Fig. 6e. Thus, this omni-compliance promotes a wider operational range of swallowing to objects’ type and size. It has been verified by effectively swallowing diverse objects, covering cylinder-extended objects (Fig. 6f), and daily objects with unique features (Fig. 6g), such as 3D-printed curved cylinders, irregular stapler, drink, fragile/heavier/soft/shape-anomalous objects, with a demonstration video (Supplementary Movie S8).

In short, the cross-linked configuration achieves the bidirectional continuous swallowing function for transportation through omni-compliance. As for the swallowable objects, the contour and size primarily depended on the grabbing performance, the minimum length of objects is mainly related to the actuator’s minimum length and connector’s height, and the weight is related to the output-force capability of longitudinal actuators during payload. Simultaneously, the omni-compliance promotes operability to adapt objects, such as oversize, irregular, and fragile objects.

**Figure 7:**
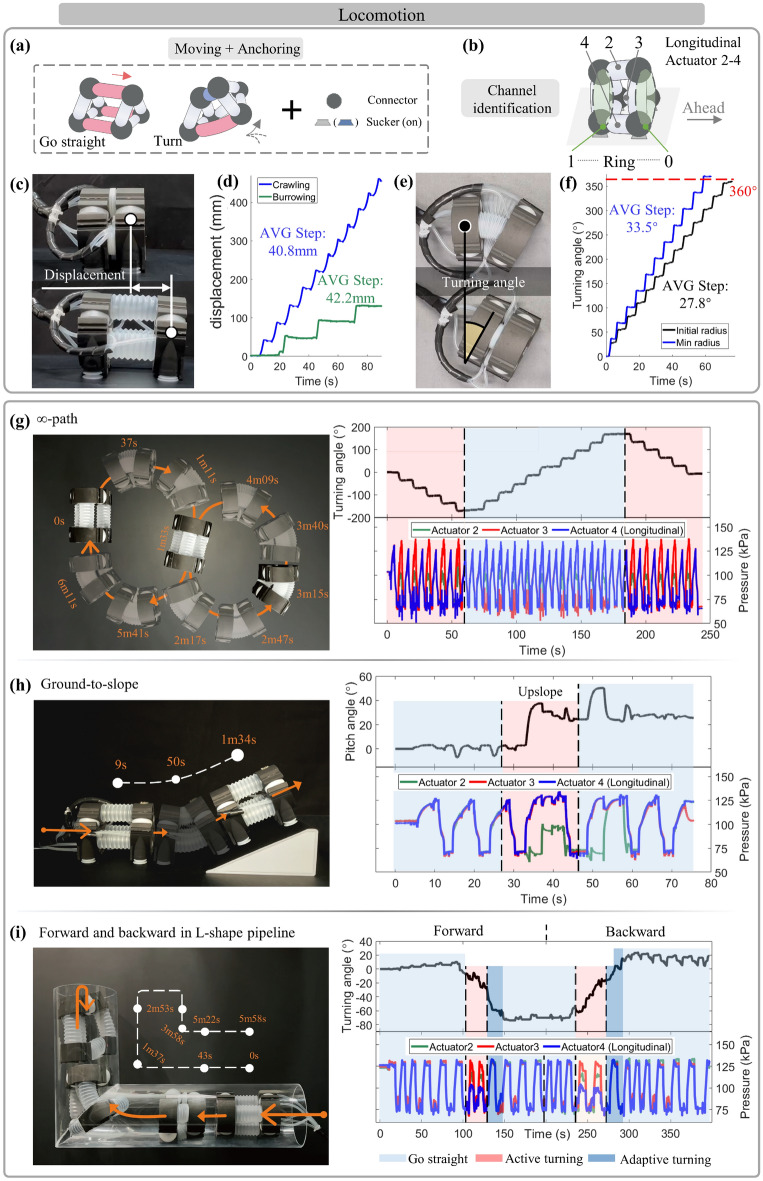
The locomotion functions in space for various terrains. (**a**) The coordination of moving and anchoring promotes the locomotion function, guided by the regular actuation (**b**) under channel identification. The collaboration of multiple longitudinal actuators allows the segment to perform the moving of (**c**) displacement and (**e**) turning angle, (**d**) with the testing average step exceeding 40 mm on the crawling and burrowing displacement, and (**f**) about 30° in turning angle. In order to validate the potential uses in complex environments, several mobile tasks were established with varying paths and terrains, covering (**g**) the ∞-path in the plane for large-range locomotion, (**h**) ground-to-slope climbing, and (**i**) forward and backward in L-shaped pipeline. Guided by the sequential-pressure regulation of the preset functions and trajectory, the segment effectively performed targeted tasks, with alternating anchoring and moving, as shown in the recorded information curves.

As for locomotion, it is achieved by the coordination of moving and anchoring within the segment to move based on the anchored location (Fig. 7a). Specifically, the coordination of multiple longitudinal actuators realizes the moving operation of displacement by elongation/contraction (Fig. 7c) and turning spatial angle by bending (Fig. 7e), in which the setting of channel identification is shown in Fig. 7b. For anchoring, replacing specific components onto the shell of the segment’s connector achieves it for the corresponding terrains, such as the suction cup used on the prototypes to cope with the flat and sloping surfaces. While in some terrains, the segment utilizes rings’ elongation to contact and fix on the surroundings (such as a pipeline). Experimentally, we first tested the prototype’s performance of displacement and turning angle. Through performing multiple loops of sequent moving, the results show the performance with the average step of crawling at 40.8 mm, the average step of burrowing at 42.2 mm (Fig. 7d), and the average step of turning angle of about 30° with a variable radius (Fig. 7f). Integrating different moving operations, it implemented the preset ∞-path, which showed the large-range spatial locomotion with mobile flexibility (Fig. 7g). With the slope or pipeline, the segment implemented to climb to the slope from the ground (Fig. 7h), as well as the operation of the forward and backward drilling in the pipeline (Fig. 7i). These verified the spatial movements and the extension to potential application for unstructured environments (Supplementary Movie S7). Thus, the proposed cross-linked actuator network brings about diverse locomotivity and potential expansibility toward diverse terrains, with the suction cup as a validation example.

### Analysis and separated/simultaneous operation of multiple functions

**Figure 8:**
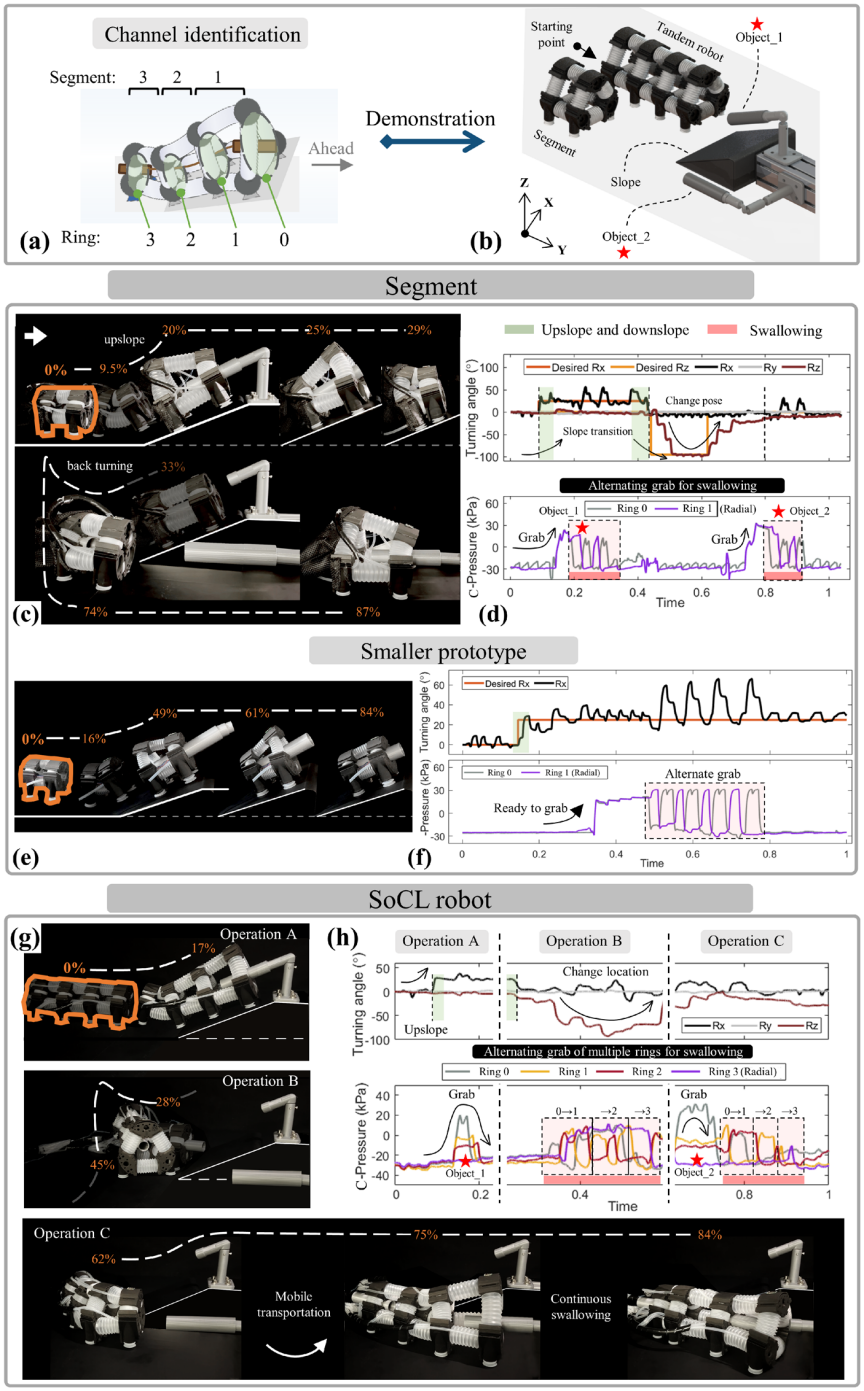
Demonstration of simultaneous multi-functions with mobile continuous transportation for objects. Covering the integration of multiple functions, (**a**) under the channel identification, (**b**) the task of mobile swallowing multiple spatial objects is set for the prototypes of the segment and the SoCL robot. (**c**) The segment fluently completed collecting objects, (**d**) with the rhythmic locomotion combined alternating grabbing to transport objects by swallowing. (**e**,**f**) The smaller prototypes performed a similar mobile operation with swallowing, verifying the universality of actuators within the proposed approach. (**g**) By splicing multiple segments modularly, the SoCL robot was constructed and implemented the same task, which exhibited the improved average step and dexterous operation of simultaneous mobile swallowing. (**h**) The turning angle of locomotion and multi-ring pressure for continuous swallowing were recorded.

By combining these functions of manipulation and locomotion, the segment shows the diversified capability of the sequent integration and simultaneous operation of multiple functions, covering grabbing, locomotion, manipulation, and transportation. These simultaneous multi-function implementations highlight the simplified approach that utilizes cross-linked configuration with omni-compliance to develop single-type actuators by a simple structure and control method. It offers a novel continuous transportation form, simultaneous operation to multiple objects in unstructured environments with either mobile manipulation or pick-and-place operation for complex tasks of potential application such as search and rescue or production-line operations. Demonstrating the performance and potentiality of the segment and the modular SoCL robot with multiple segments, different testing tasks were presented, including: (1) mobile grabbing, manipulation, and continuous transportation of two targeted objects in space (Fig. 8b), and (2) manipulation of multiple objects in different locations with continuous transportation.

Regarding testing task 1, the segment walked straight to climb up the slope and retrieved the high-altitude object. It then transported the object internally that captured it by continuous swallowing. The segment moved backward to climb down the slope, turned angle to move to other positions for capturing other objects continuously (Fig. 8c). The pressure of rings was recorded to display the actuation state for grabbing and swallowing during the entire process, following the channel identification in Fig. 8a. The corresponding curve of the real-time turning angle has the same sequence to display the synchronous spatial movement (Fig. 8d), accompanied by the process video as shown in Supplementary Movie S1. This demonstration exhibits the capability of mobile manipulation in large-scale space without interruption during the sequential operation of multiple objects. The unique mobile swallowing to continuously transport objects introduces novel operating forms and improves the operational flexibility for complex tasks and potential applications.

The proposed approach has the potential universality to the actuator, with a low scale limit, which has been verified by a smaller segment prototype (including the performance tests and model verification corresponding to the normal-size prototypes, as shown in Supplementary Fig. S9). With a similar process, the smaller segment straight walked to climb up the slope to grab and swallow an object (Fig. 8e). This demonstrated the mobile-manipulation feasibility, in which the circular-ring pressure is alternating changed for swallowing accompanied by moving (Fig. 8f). The task’s effective operation by smaller prototypes verified the proposed approach’s universality, which allowed for multifunctional capability even changing the actuator.

Stacked by multiple segments, the SoCL robot modularly extends the operable scale and flexibility and promotes the capability of simultaneous multi-object operation, which broadens the continuous-transportation application. For demonstration and validation, we tested the performance of the SoCL robot in processing task 1. With a larger average step by simultaneous three-segment movement, the SoCL robot completed the mobile process in fewer steps. During the similar upslope to capture an object, downslope to change position, and transport another object, the SoCL robot continuously transported objects within simultaneous locomotion (Fig. 8g) (Supplementary Movie S2). The recorded pressure of the circular ring supports this operation of the SoCL robot to continuously transport objects ring by ring, accompanied by the displacement and turning angle of locomotion (Fig. 8h). This shows the stackable expandability of configuration and the flexibility of novel operations, with the capability of continuous transportation of objects in complex/unstructured environments, for potential applications such as search and rescue.

**Figure 9:**
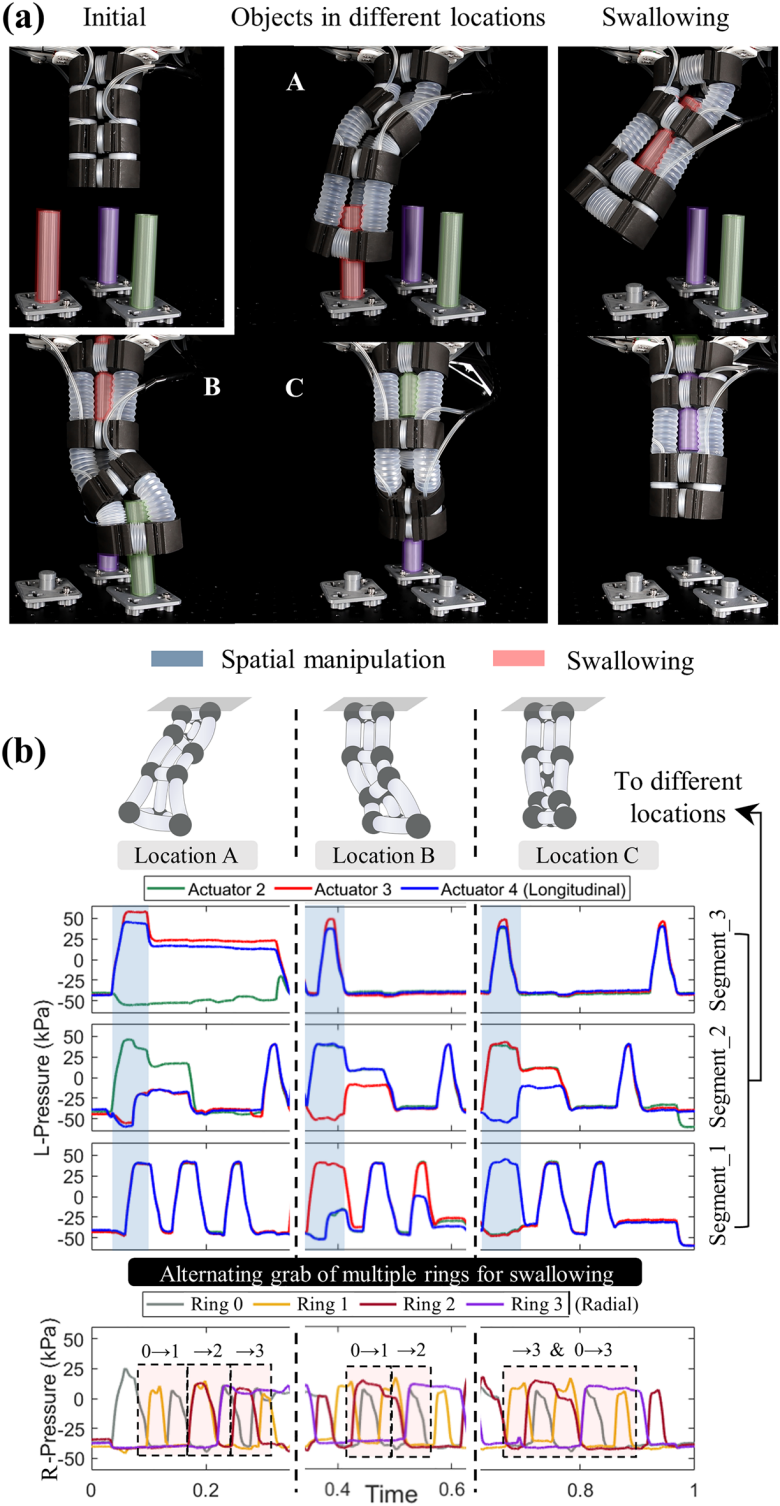
Demonstration of simultaneous multi-functions with continuous transportation of multiple objects in different locations. (**a**) With the initial lifting state, the SoCL robot exhibited arm-liked states with simultaneous multi-functions. It performed the continuous-operation task of swallowing and manipulating multiple objects simultaneously. With the recorded pressure as shown in (**b**), the prototype of the SoCL robot has the manipulation ability to reach spatial locations while integrating the alternating grabbing of multiple rings for rhythmic simultaneous swallowing to continuously and simultaneously transfer multiple objects.

To explore the SoCL robot’s capability of continuous transportation of simultaneous multi-objects with the fixed base, we set up task 2 for the SoCL robot to grab and transport multiple objects in different locations. Similar to a soft arm, the SoCL robot achieves manipulation by separating movements of the robot’s segments to arrive at the spatial location within the workspace. Through the novel continuous-transportation operation to grab and swallow the object, the SoCL robot changed to the next targeted location during the continuous swallowing for simultaneous multiple operations. By constantly changing the location of the robot-head segment to grab objects, the SoCL robot implemented the continuous transportation behavior of simultaneous multi-objects (Fig. 9a) (Supplementary Movie S3). The recorded pressure of rings is alternately regulated to transport objects, and the pressure processes of different-object operations are integrated to form the overlapping staggered fluctuations in the curve during changing position (Fig. 9b), to support this operation of simultaneous multi-objects. The effective execution of task 2 by the SoCL robot validates the novel continuous-transportation mode, which can be applied in simultaneous multiple objects for potential applications such as production-line operations.

Thus, the scale extendibility of the cross-linked actuator network and its potential universality for actuators expand the generalizability and application scope of the approach, allowing the customized design of segment and SoCL robot for diversified tasks. In which the robotic average-step performance mainly depends on the performance of the actuator’s active actuation. Besides, this cross-linked configuration promotes the single-type actuators to realize the manipulation capability of simultaneous multi-functions and multi-objects by a simplified approach, bringing a brand-new operation form of continuous transportation and improving the variety of soft robot designs for complex potential applications.

## Discussion

The compliance inherent in soft robots offers them not only continuous and dexterous movements, but also high adaptability to unstructured environments. These characteristics make the soft robot behave very similarly to the biological systems. In this work, we demonstrated that high functionality of the soft robotic system could be obtained with a cross-linked actuator coordination mechanism by taking full advantage of the inherent compliance. With this mechanism, we introduced a cross-linked actuator network where two or more identical soft actuators are crossed and linked to each other generating adaptive actuation, forming such modules as a 2D ring and a 3D segment for multiple degrees-of-freedom movements. This brings new motion patterns with simultaneously radial-axial movements that combine radial inflation with the conventional axial linear and bending movements.

To gain insight into the actuator coordination mechanism and offer general guidance for soft robotic design, we modeled the relationship between the performance and design parameters. It was elaborated that spatial kinematics in simultaneously radial-axial movements, together with the static radial forces, are mainly decided by the numbers and dimensions of actuators. Following this rule, the coordination and trade-offs between the radial and axial movements were further analyzed for various motion patterns. These developed models are essential for the modular design of function composition. Further, we introduced finite automata that complete the function composition by sequencing the various modular motions, allowing the robot to maintain this multi-functionality. It serves mainly as a bridge that receives the designated function according to the application request and generates the motion sequences of the modules to send to the actuation system. This approach enables the multi-functionality of soft modular robots with a relatively simple control strategy. Following this spirit, we proposed a controller with a high-level layer for function composition that can be tailored to the task and a low-level layer for pressure control with multiple circuits independently controlled. The proposed control strategy shows high simplicity and extensibility owing to the concise actuation set composed of one-type soft actuation modules. This complete methodology, from the design of a cross-linked network and modeling of coordinated kinematics to a modular control strategy, offers comprehensive guidance for the full customization of high-functionality soft robotic systems.

Following the developed methodology, a soft cross-linked modular robot was developed to demonstrate simultaneous multi-functions. Here, a diversity of basic functions was analyzed, including grabbing, swallowing, manipulation, and locomotion. Our approach offered three grabbing modes to grab different shapes of objects adaptively. In particular, one of the modes could firmly wrap the objects resisting a pulling force exceeding 200N. Also, the new swallowing function was verified by continuously swallowing objects with variable shapes. The analysis of these functions further enables the combination of simultaneous multi-functions. A novel continuous-transportation mode was demonstrated, for which multiple objects could be simultaneously transported in unstructured environments with either mobile manipulation or pick-and-place operation. The experimental results showed that the robot in mobile manipulation could locomote on flat and sloping ground to transport several objects simultaneously with a combination of grabbing, locomotion, and swallowing. Besides, the robot in pick-and-place operation could collect the objects distributed on the desk and simultaneously transport them with a combination of grabbing, manipulation, and swallowing. This work extends the use of compliance to coordinate actuation movements of the cross-linked soft actuators, filling the gap between the understanding of soft robotic actuator coordination mechanism and the high biological functionality, and paving the way to new soft robotic designs for many robotic applications, such as industrial manipulation and unstructured environmental exploration.

Future work will explore the proposed approach’s potential applications, as well as further developments in the design of various soft robotic systems. The modeling and control of robotic kinetics will also be investigated for operation in various scenarios, such as locomotion in rugged terrain and manipulation with heavy objects.

## Methods

### Fabrication of prototypes

The bellows’ end was cut with a hole about 10 mm in diameter and then aligned and hot-glued to the protruding channel port on the 3D-printed connector (nylon, selective laser sintering). The glue will flow into the inside of the bellows end through the gap between the hole and the channel port, creating adequate air tightness. The connector is designed with a built-in gas channel and air outlet, linking the radially adjacent bellows, while the channel of the longitudinal actuator is independent. It prototyped the configurations of ring and segment by different-scale bellows, following the most simplified case of N = 3. Then, the soft trachea and pressure sensors are linked to the air outlet for actuation.

### Replaceable components of the segment

The connector of prototypes has a slot on the outside that can be used to equip different components by interference fit and hot glue. For the crawling of prototypes, we chose the shell with suction cups, with an adhesive net pocket behind the segment when showing the mobile continuous swallowing.

### Experimental setup and methodology

#### Pneumatic characterization

For pneumatic actuation, we built a steady supply device of air (Supplementary Fig. S2a), providing positive pressure 60 kPa and negative pressure − 60 kPa to the pneumatic actuation system. This device had a compressor pump (DET750-5L, DAERTUO Inc.) and a vacuum pump (v750, Fujiwara Inc.).

#### Experimental sensors, signal acquisition, and processing

Experimental information mainly includes the changes in output performance and state during the motion and interaction process by pneumatic actuation, covering displacement, force, rotation angle, etc. Therefore, we used multiple sensors to obtain raw data, including the pressure sensor (XGZP6857A, ± 100 kPa, CFSensor Inc.) for internal pressure of actuators, the laser sensor (HG-C1100, Panasonic Inc.) for displacement, the one-dimensional force sensor (AR-DN103, 0-100N & 0-500N, 0.2%, Arizon Inc.), and IMU (MPU6050, InvenSense Inc.) for angle.

All analog sensing data is collected by the ADC module board (AD7606, ADI Inc.) and transmitted to the microcontroller (STM 32 F103ZET6, ST Microelectrons Inc.) along with the IMU transmitted through serial communication to record the changes of the monitoring quantity in real-time. Data is processed on MATLAB (MATLAB R2018b, MathWorks, USA) to obtain analysis results and figures.

#### Experiments of the bellows

We test the linear displacement and output force under pneumatic actuation. As for displacement experiment, the bottom of the bellows was horizontally mounted on the 3D-printed base (Supplementary Fig. S5a), while the end of the bellows was linked with a plate on bilateral slide rails to limit the motion except for longitudinal direction. The bellows push the plate to move by reciprocating elongation and contraction, synchronously monitoring plate displacement and actuator pressure. As for the output force experiment, the plate was fixed in a specific position to limit the bellows’ length by connecting a screw rod with a self-locking motor. The corresponding output force was recorded with synchronous pressure data during the changed pressure.

The lateral compliance is a part of the actuator’s omni-compliance, represented by the lateral stiffness $k_{r}$. In the calibration experiment, the bellows is vertically fixed on the plane (Supplementary Fig. S5c), where the top is free and connects a string. The bellows undergoes the compliant lateral deformation by driving the motor and screw rod to uniformly pull the string. The displacement and force of the screw rod are approximated as the lateral deformation and force of the actuator, and then the lateral stiffness can be obtained by fitting the slope.

#### Experiments of the ring

We test the radial displacement and output force under the pneumatic actuation to verify the model and show the motion characteristics. As for displacement experiment, the ring is horizontally installed on three sliding rails, wherein the slide rails are arranged circularly with equal angle spacing, and the sliding direction is outward from the center of the circle. This ensures unobstructed radial motion of the ring and restricts movement in other directions. Assuming the center of the ring keeping unchanged during movement, the ring undergoes reciprocating elongation and contraction, and the radius is measured by the laser sensor, which is fixed on the side and aimed at the connector of the ring.

As for output force experiment, some grabbed balls with different radii are constructed, consisting of two printed pieces without contact and connected to both sides of the force sensor (Supplementary Fig. S6a). When the ring performs contraction by negative pressure to grip the ball, the pressure and grasping force are recorded in real-time, covering multiple cases of different ball radii.

#### Anti-pull-out force of multimodal grabbing

The anti-pull-out force resists the grabbed objects being forcibly pulled off, representing the grabbing stability of the segment. In the test, the motor drives the screw to move the ball-liked end to a specific center distance $D_{L}$. The grabbing ring contracts further to keep constant pressure for grabbing. Next, the motor moves uniformly in reverse to pull outward the ball-liked end axially (Supplementary Fig. S6b). During the process, the pull force is recorded, which curve peaks at structural pull-off (the ring is stretched) or destructive defense (the structure is damaged or the motor is slipping). Besides, the experiments of different cases are covered by adjusting the center distance $D_{L}$ and the contraction pressure, repeating the entire process.

The anti-pull-out ability in an angled direction donates to stable grabbing in different cases. By replacing the fixing bases with inclination, the segment is installed at a specific angle (Supplementary Fig. S3c) and then performs the above process, promoting a similar experiment within changed direction cases.

#### Segment’s variable-radius bending

The experiments of variable-radius bending are performed to test the maximum angle in different cases, covering constant $r_{d} = 0$ and changed $r_{d} \ne 0$. This work measures the maximum angle during a cyclic extreme-pressure sequence of longitudinal actuators, taking the average value.

As for $r_{d} = 0$, the tracheas of the upper and lower rings are merged and changed synchronously. As for $r_{d} \ne 0$, the radius of the upper and lower rings is adjusted separately to set a specific case of $r_{d}$. During the experiment, the segment’s top is connected to a rigid plate with IMU to collect the angle data.

#### Functional performance and demonstration

The processes of testing performances and demonstration tasks of the segment and robot are guided by the corresponding targeted pressure sequences (obtained by presetting or the solution solved by the control strategy) and be implemented by the pneumatic actuation. The temporal raw data were recorded, including the pressure, spatial turning angle, and optional displacement.

## Supplementary Information

Supplementary Information.

## Data Availability

The data supporting the findings and detailing the studies are available from the first author or corresponding author upon request.
