# Supplementary material for: Omnidirectional compliance on cross-linked actuator coordination enables simultaneous multi-functions of soft modular robots
Source: Sci Rep. 2023 Jul 26;13:12116. doi: 10.1038/s41598-023-39109-2 (PMC10372032; doi:10.1038/s41598-023-39109-2)
Supplement: Supplementary file 1 — Supplementary Information. [file 41598_2023_39109_MOESM1_ESM.zip › Supplementary_informantion_revision/supplementary_Note_revision.pdf]

Supplementary Information

Omnidirectional compliance on cross-linked actuator coordination enables simultaneous multi-functions of soft modular robots

Zhonggui Fang[1,2], Yige Wu[2], Yinyin Su[2,3], Juan Yi[1,2*], Sicong Liu[2], Zheng Wang[2*]

1. Shenzhen Key Laboratory of Intelligent Robotics and Flexible Manufacturing Systems, Southern University of Science and Technology, Shenzhen, China
2. Department of Mechanical and Energy Engineering, Southern University of Science and Technology, Shenzhen, China
3. Department of Mechanical Engineering, The University of Hong Kong, Central And Western District, Hong Kong SAR, China

Supplementary note 1: Derivation of the model of actuator state in 2D ring
Supplementary note 2: Obverse kinematic model of the segment and SoCL robot
Supplementary note 3: Inverse kinematic model
Supplementary note 4: Simulation of the kinematic model
Supplementary note 5: Supply device of air and the performance of angle controlling

Supplementary Movie S1: Mobile continuous swallowing of the segment
Supplementary Movie S2: Mobile continuous swallowing of the SoCL robot
Supplementary Movie S3: Simultaneous multi-objects swallowing of the SoCL robot
Supplementary Movie S4: The motion characteristic of the actuator, ring and segment
Supplementary Movie S5: Anti-pull-out performance of grabbing
Supplementary Movie S6: Swallowing
Supplementary Movie S7: Locomotion
Supplementary Movie S8: Swallowing function toward diverse objects

Supplementary Notes

Supplementary note 1: Derivation of the model of actuator state in 2D ring

Based on the equation (1.1) in the model of actuator state in 1D, we analyze the geometric relationship between 2D ring and 1D actuator (Supplementary Fig. 1a), which can be expressed as:

$$\Delta \mathrm{r} = \Delta l / (2\cos(\pi/2 - \pi/N)) \tag{S1.1}$$

$$F_r = 2F_l \cos(\pi/2 - \pi/N) \tag{S1.2}$$

Substituting these relationships into equation (1.1), the model of actuator state in 2D can be got as equation (1.2).

Supplementary note 2: Obverse kinematic model of the segment and SoCL robot

The segment has the unique bending with variable radius. Based on the mentioned model of actuator state, we get each length of the longitudinal actuator and radius of 2D ring from the sensed pressure. To analyze the obverse kinematic model for the configuration state and workspace, the segment was divided evenly into n parts. Within each part, it has the same radius of upper and lower rings, and different radius between adjacent parts. The parameters for the m-th part of the segment is:

$$\begin{aligned} l_{mi} &= l_i / n \\ r_m &= r_1 + m(r_2 - r_1)/n \end{aligned} \tag{S1.3}$$

where l_{mi} is the length of the i-th longitudinal actuator with i=1,2,3, and r_m is the radius.

Assuming piecewise constant curvature (PCC), the forward kinematic model of the m-th part can be expressed as

$$\begin{aligned} \alpha_m &= \frac{2\sqrt{l_1^2 + l_2^2 + l_3^2 - l_1 l_2 - l_1 l_3 - l_2 l_3}}{3nr_1 + 3m(r_2 - r_1)} \\ \phi_m &= \tan^{-1}[\frac{\sqrt{3}(l_3 - l_2)}{l_3 + l_2 - 2l_1}] \\ l_m &= \frac{l_1 + l_2 + l_3}{3n} \end{aligned} \tag{S1.4}$$

where α_m is the curvature angle around deflection axis, ϕ_m is the deflection angle around the z-axis and l_m is the length of curvature center.

Then, through integrating of multiple parts, we have the configuration state of the segment as:

$$\alpha = \int_1^n \alpha_m dm = \frac{2\sqrt{l_1^2 + l_2^2 + l_3^2 - l_1 l_2 - l_1 l_3 - l_2 l_3}}{3(r_2 - r_1)} \ln \frac{r_2}{r_1}$$
$$\phi = \int_1^n \phi_m dm = \tan^{-1}[\frac{\sqrt{3}(l_3 - l_2)}{l_3 + l_2 - 2l_1}] \quad \text{(S1.5)}$$
$$l = \int_1^n l_m dm = \frac{l_1 + l_2 + l_3}{3}$$

Where α, ϕ, and l is the configuration space of the segment.

Furthermore, symbolized the workspace, the transition matrix U_k^{k+1} from the top of the k-th segment to the top of next one can be expressed as:

$$U_k^{k+1} = \begin{bmatrix} \Phi_k^{k+1} & o_{kt}^{(k+1)t} & 0 \\ 0 & 1 & 0 \\ 0 & 0 & R_{k1}^{k2} \end{bmatrix} = \begin{bmatrix} \Phi_k^{k+1} & o_{kt}^{(k+1)b} & 0 \\ 0 & 1 & 0 \\ 0 & 0 & R_{k1}^{k2} \end{bmatrix} \begin{bmatrix} \mathrm{I} & [0,0,h]^T & 0 \\ 0 & 1 & 0 \\ 0 & 0 & 1 \end{bmatrix}$$
$$= \begin{bmatrix} c_\phi^{\,2}(1-c_\alpha)+c_\alpha & c_\phi s_\phi(1-c_\alpha) & s_\phi s_\alpha & l(1-c_\alpha)c_\phi/\alpha + s_\phi s_\alpha h & 0 \\ c_\phi s_\phi(1-c_\alpha) & s_\phi^{\,2}(1-c_\alpha)+c_\alpha & -c_\phi s_\alpha & l(1-c_\alpha)s_\phi/\alpha - c_\phi s_\alpha h & 0 \\ -s_\phi s_\alpha & c_\phi s_\alpha & c_\alpha & l s_\alpha/\alpha + c_\alpha h & 0 \\ 0 & 0 & 0 & 1 & 0 \\ 0 & 0 & 0 & 0 & R_{k1}^{k2} \end{bmatrix} \quad \text{(S1.6)}$$

with the radius state $R_{k1}^{k2} = \begin{bmatrix} r_{k2} & 0 \\ 0 & r_{k1} \end{bmatrix}$, where the r_{k1} and r_{k2} are the radius of the upper and lower rings in the k-th segment. Where Φ_k^{k+1}, $o_{kt}^{(k+1)t}$ is the rotation matrix and displacement matrix. s_α, c_α, s_ϕ, c_ϕ is respectively the trigonometric function (sin & cos) of the angle α and ϕ, and h is the thickness of the connector in the segment.

Splicing multiple segments to build the soft cross-linked (SoCL) robot by (Supplementary Fig.1b), the transition matrix of the SoCL robot can be obtained as:

$$U_1^i = \prod_2^i U_{k-1}^k \quad \text{(S1.7)}$$

accompanying with the structural constraint $r_{k1} = r_{(k-1)2}$ for every k of segment.

Supplementary note 3: Inverse kinematic model

We constructed the inverse kinematics model for the 3D segment, to calculate the analytical solution of actuator pressure group $\{P_{l1}, P_{l2}, P_{l3}, P_{c1}, P_{c2}\}$ from the desired position and posture state $\{w_x, w_y, w_z, \alpha_d, \phi_d, l_d, r_{2d}, r_{1d}\}$ of the segment. Firstly, the

pressure of circular rings $\{P_{c1}, P_{c2}\}$ can be obtained through the inverse function of the equation (1.2).

To ensure the existence of analytical solutions, the desired input is necessary to be inside the workspace based on the forward kinematics model. Therefore, we have the functions of desired position $\{w_x, w_y, w_z\}$ as:

$$\begin{aligned} w_x &= 3l_d c_{\phi_d}(1-c_{\phi_d})/\alpha_d \\ w_y &= 3l_d s_{\phi_d}(1-c_{\phi_d})/\alpha_d \\ w_z &= 3l_d s_{\alpha_d}/\alpha_d \end{aligned} \tag{S1.8}$$

and the desired longitudinal actuator length $\{l_1, l_2, l_3\}$ have the following relationship with the desired configuration state $\{\alpha_d, \phi_d\}$ as:

$$\begin{aligned} \tan\phi_d &= \sqrt{3}(l_3-l_2)/(l_3+l_2-2l_1) \\ \alpha_d &= \frac{2\sqrt{l_1^2+l_2^2+l_3^2-l_1l_2-l_1l_3-l_2l_3}}{3(r_2-r_1)}\ln\frac{r_2}{r_1} \end{aligned} \tag{S1.9}$$

Through the equations (S1.8) and the formula of the curvature center length in (S1.5), we can obtain the relationship between the length of longitudinal actuators:

$$\begin{aligned} l_2 &= (w_z\alpha_d/s_{\alpha_d}-3l_1)(\sqrt{3}-\mathrm{t}_{\phi_d})/(2\sqrt{3})+l_1 \\ l_3 &= (w_z\alpha_d/s_{\alpha_d}-3l_1)(\sqrt{3}+\mathrm{t}_{\phi_d})/(2\sqrt{3})+l_1 \end{aligned} \tag{S1.10}$$

Then, the desired length of longitudinal actuators $\{l_1, l_2, l_3\}$ were solved, through substituting the relationship of equations (S1.10) into the equation (S1.9) and simplifying. It is shown as:

$$\begin{aligned} l_1 &= w_z\alpha_d/s_{\alpha_d}+(-1)^j\frac{\alpha_d(r_{2d}-r_{1d})t_{\phi d}}{\ln(r_{2d}/r_{1d})\sqrt{1+t^2_{\phi d}}} \\ l_2 &= l_1+(-1)^{j+1}\frac{\alpha_d(r_{2d}-r_{1d})(3t_{\phi d}-\sqrt{3})}{2\ln(r_{2d}/r_{1d})\sqrt{1+t^2_{\phi d}}} \\ l_3 &= l_1+(-1)^{j+1}\frac{\alpha_d(r_{2d}-r_{1d})(3t_{\phi d}+\sqrt{3})}{2\ln(r_{2d}/r_{1d})\sqrt{1+t^2_{\phi d}}} \end{aligned} \tag{S1.11}$$

where j represents the quadrant state of the desired deflection angle ϕ_d. We have $j=0$ when $\phi_d \in [\frac{\pi}{2},\pi]\cup[-\frac{\pi}{2},-\pi]$, and conversely $j=1$ when $\phi_d \in [-\frac{\pi}{2},\frac{\pi}{2}]$. Thus, the expected pressure of the longitudinal actuators $\{P_{l1}, P_{l2}, P_{l3}\}$ can be obtained by the inverse operation of equation (1.1) as $P_{li} = h^{-1}(l_i)$ (i=1,2,3), with the hypothesis that payload is zero.

So far, the required actuator-pressure group $\{P_{l1}, P_{l2}, P_{l3}, P_{c1}, P_{c2}\}$ for the desired state $\{w_x, w_y, w_z, \alpha_d, \phi_d, l_d, r_{2d}, r_{1d}\}$ was solved, which is the inverse kinematic model of the 3D segment with unique variable-radius bending.

As the shown in Supplementary Fig.1f, the verification of the inverse kinematic model was performed, using different inputs of the preset expected trajectories, including spiral, square, and circle in space. Through calculating the solutions from model and comparing to the target (in the method), the result shown the consistency, which demonstrated the accuracy and effectiveness of the inverse kinematic model.

Supplementary note 4: Simulation of the kinematic model

To explore the influence of the segment's workspace, we simulated the workspace with the changed radius distance r_d, based on the proposed observe kinematic model (Supplementary Fig.1c). The schematic diagram shows the decreasing workspace as the r_d increase, which promote the potential of grabbing during manipulation and locomotion. To quantify the influence of changed workspace, the volume of workspace was estimated by simulation, which show the acceptable reduction, with the minimum retention of 75% (Supplementary Fig.1d).

With the kinematic model of the SoCL robot, we explored the workspace of robot with the example of three segments (Supplementary Fig.1e). The schematic diagram shows the gradual-increasing workspace of segments, which is beneficial for expanding applications.

The inverse kinematic model was verified by simulation. Setting the preset-input discrete trajectory, covering spatial square and circle shapes, the estimated solution from the kinematic model was solved to compare the input. The result's consistency demonstrated the model's effectiveness (Supplementary Fig.1f).

Supplementary note 5: Supply device of air and the performance of angle controlling

We set up the air supply device to ensure adjustable and stable air source for pneumatic actuation system, covering positive and negative pressure (Supplementary Fig.2a). Due to this equipment foundations, we achieved the precise and high-frequency motion capability of prototypes, with experimental verifications of controlling bending angle of the segment (Supplementary Fig.2b). Adjusted reciprocally the targeted rotation angle for the segment and performed multiple experiments with different frequency, the prototype of the segment responded the desired angle stably even the frequency reaches 1Hz.

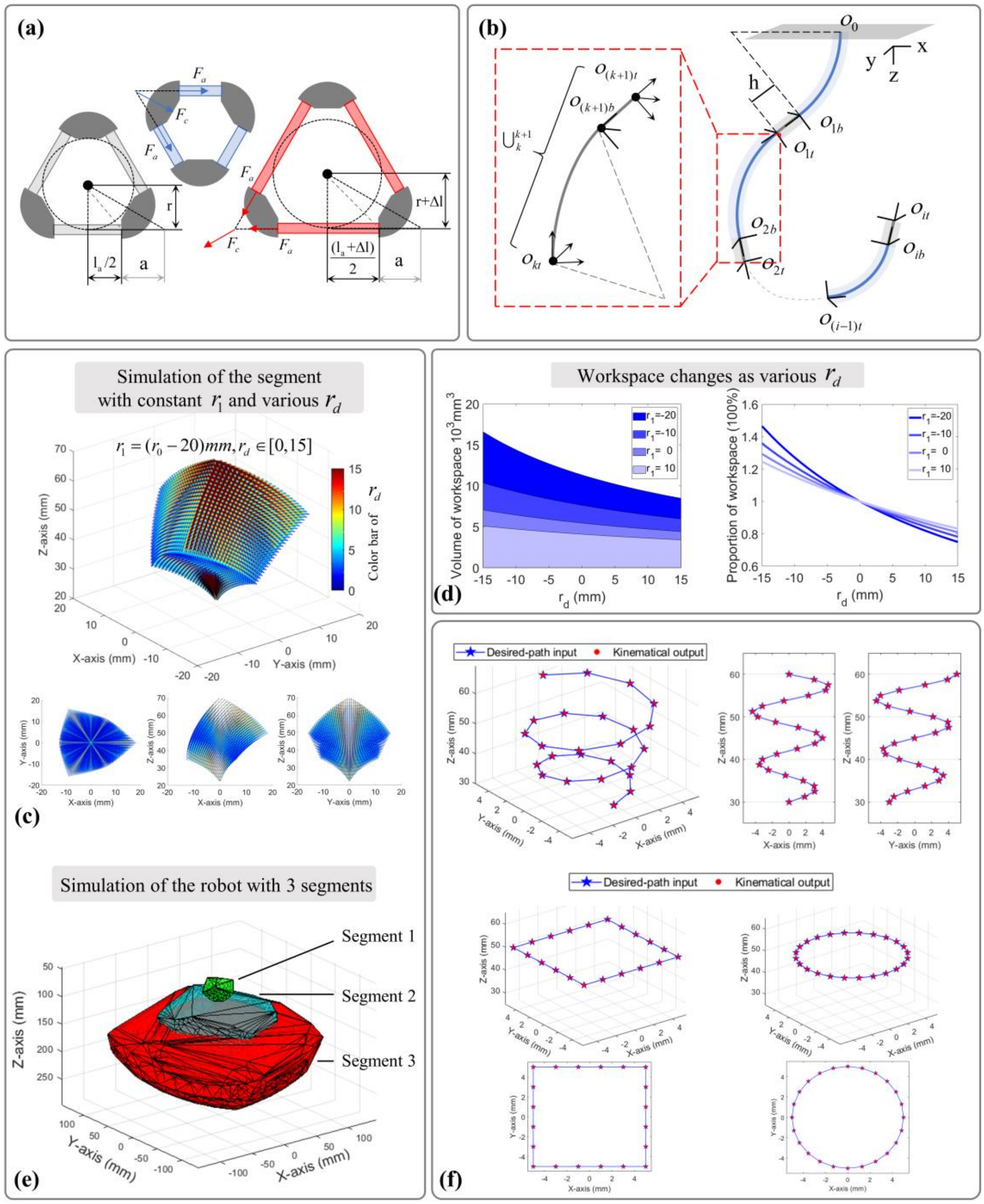

Supplementary Fig. S1. The derivation, simulation, and verification of modeling. (a) The radial motion of the ring was analyzed by geometric derivation from the actuator state of linear motion. Building the bidirectional kinematic model with obverse and inverse one, (b) the spatial state of the SoCL robot can be estimated, with the derivation of transformation matrix. (c) We simulated the workspace of the segment's unique variable-radius bending, based on the proposed kinematic model with the changed radius distance r_d. Besides, to explored the influence of workspace by the changed r_d, (d) we analyzed the changed volume of workspace. (e) The workspace of the SoCL robot was simulated with the example of three segments. (f) The inverse kinematic model was verified through comparison of the model solution and the preset-input discrete trajectory, covering spatial square and circle shapes.

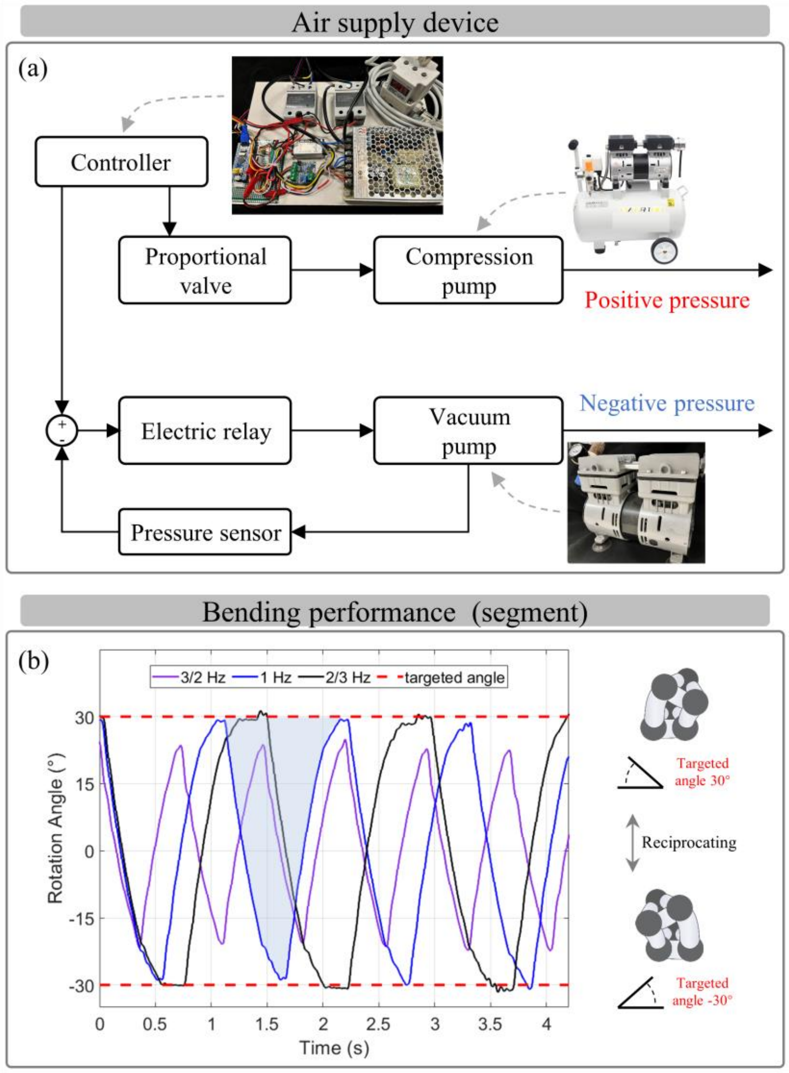

Supplementary Fig. S2. The air supply device and the achievable actuation performance. (a) Based on the pump system, the stable air supply for the pneumatic actuation system was achieved, covering positive pressure and negative pressure. With the equipment foundation, (b) the segment shown accurate and high-frequency response, while the experimental test of bending achieved 1Hz frequency to the targeted-angle setting of ±30°, with a precise angle control.

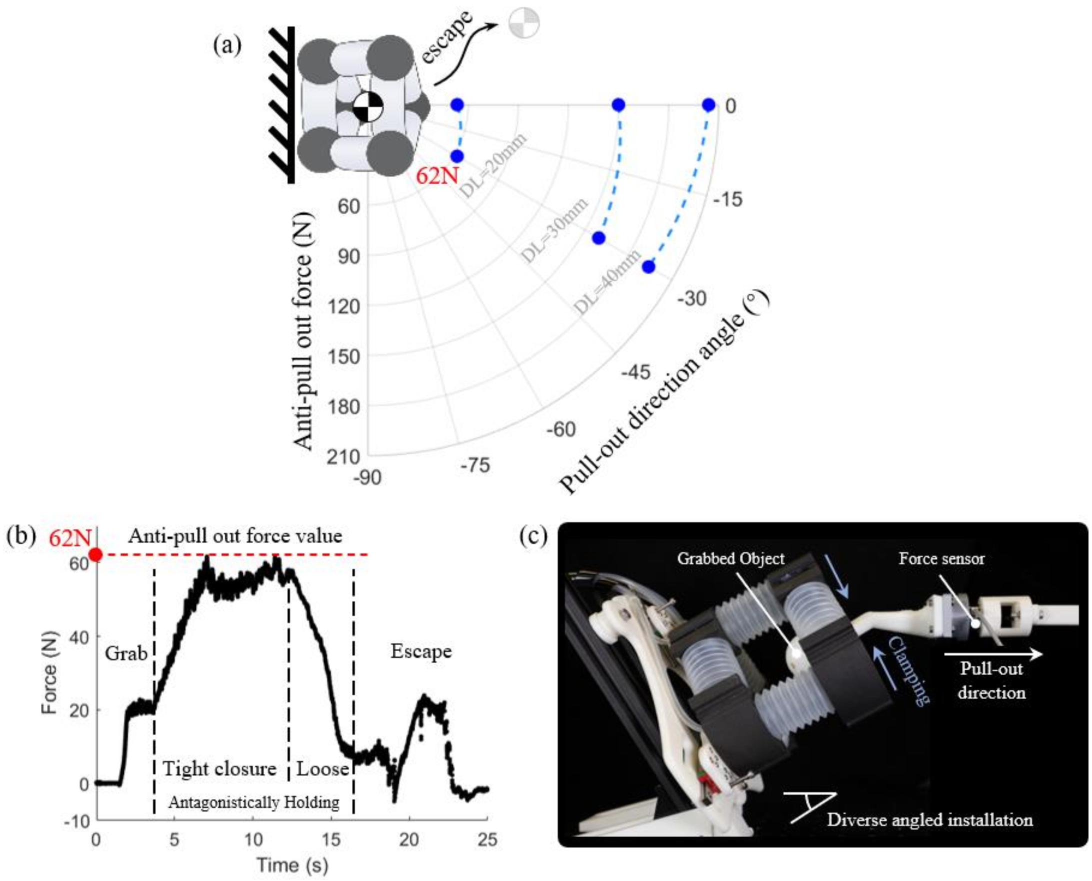

Supplementary Fig. S3. Oblique anti-pull-out performance of the segment. Installed at a specific angle (c), the segment conducted the anti-pull-out force experiment to explore the lateral stability of grabbing. With the similar experimental process, the gripping force of the segment exhibits a similar peak-like curve (b). The result (a) shown the commensurate anti-pull-out performance of longitudinal and angled direction, with approximate value in multiple cases with the same DL. The maximum anti-pull-out force is about 200N at the modal of wrapped grab, exhibiting the equivalent performance to axial stability.

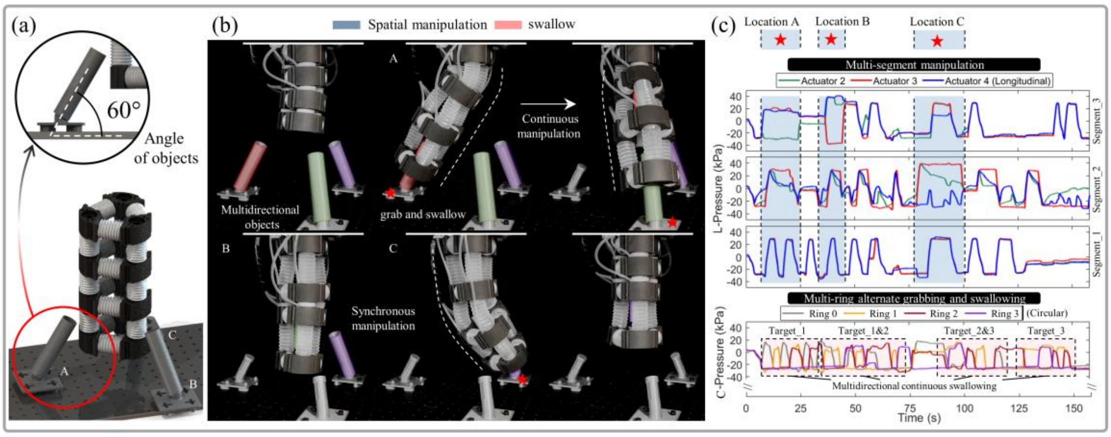

Supplementary Fig. S4. The capability of continuous swallowing grab in the SoCL robot, which is applies to angled-install objects. For verification, (a) the scene was arranged with multiple angled objects, and (b) the SoCL robot operated effectively grabbing task with continuous swallowing process, guided by sequential targeted pressures. (c) The corresponding pressure was recorded during the process, which exhibited the expected steps, with the alternating ring motion for grabbing and swallowing, and the longitudinal actuator cooperation for bending to the sequential targeted locations. The process is shown in the Supplementary Movie S3.

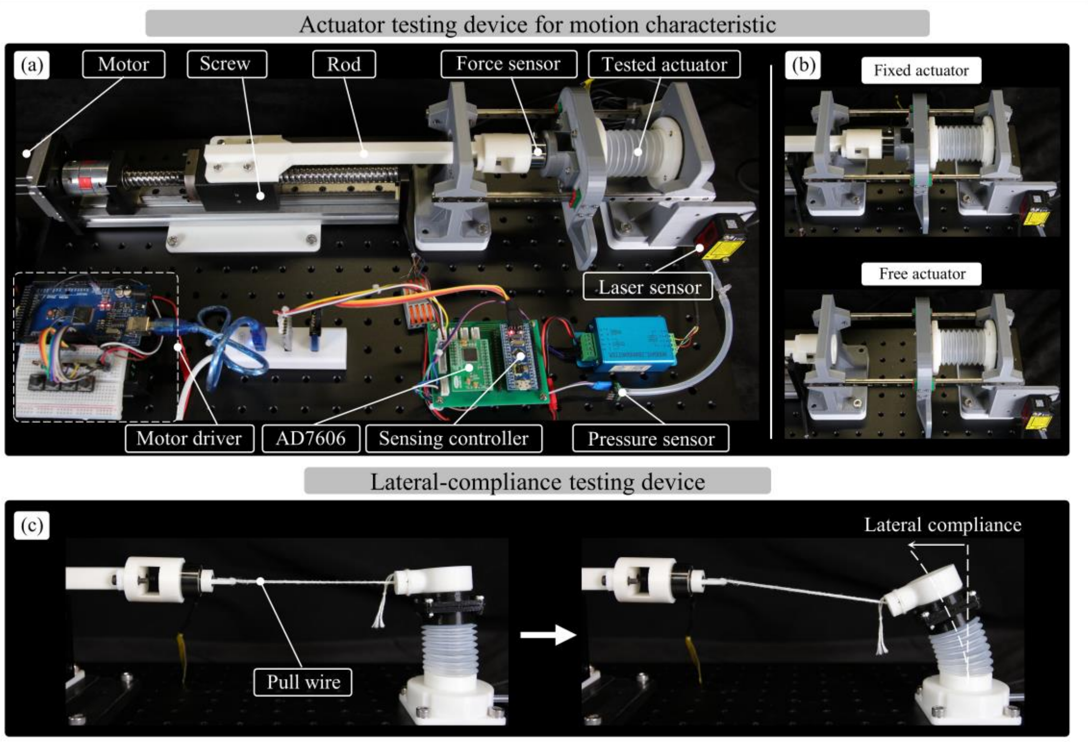

Supplementary Fig. S5. The experimental devices of actuator-testing and the data acquisition system. (a, upper) The longitudinal testing device of actuator was built by the motor-screw system, (b) provided two types of actuator state with fixed and free one, to separately explore motion characteristic of longitudinal output force and displacement. (c) As for lateral compliance, the pull-wire was used to act on actuator. (a, lower) The collection system for the experimental data with controller.

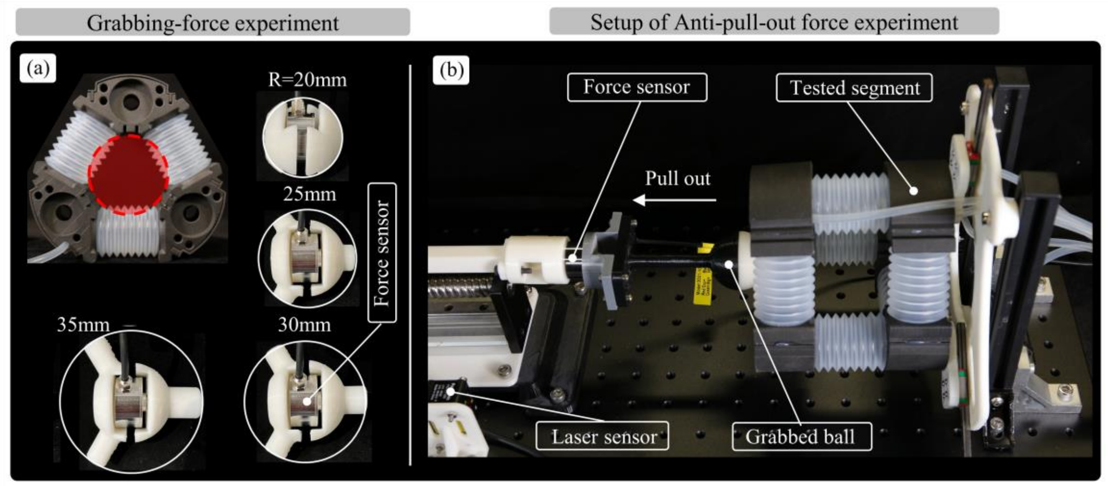

Supplementary Fig. S6. The grabbing force testing devices of the ring and the segment. As for circle grabbing, (a) multiple grabbed ball was built with different radius, and the force sensor was sandwiched to obtain experimental data when the ring grab balls. To explored the axial anti-pull-out performance, (b) the segment was horizontally installed, and the ball-liked end of the rod can be driven by motor to move uniformly. Equipped with sliding rails, the segment can be contraction to grab object.

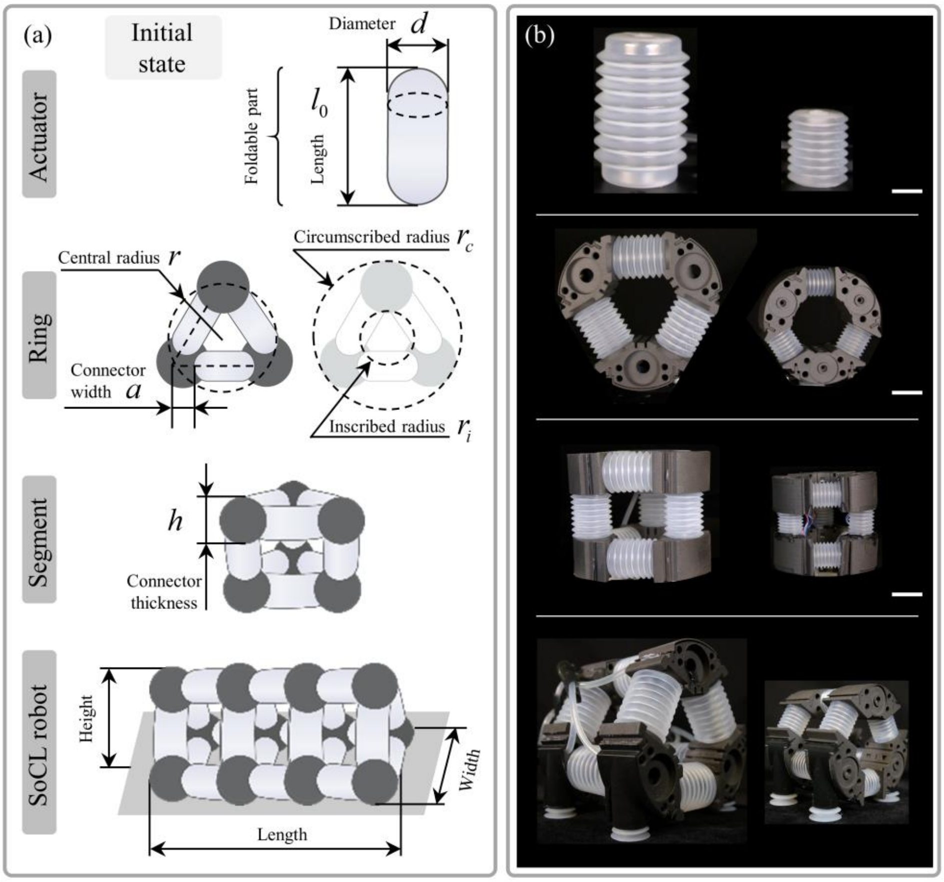

Supplementary Fig. S7. The schematic diagram of parameters (a) and prototypes (b) of configurations in different dimensions, covering actuator unit, ring, segment, and the SoCL robot, while the prototypes were built by soft bellows. The specific values are shown in table S1.

Table S1: The design parameters of prototypes and the physical quantities values

	Length l_0 (mm)	Section diameter (mm)	Calibrated longitudinal stiffness (N/mm)	Calibrated cross-sectional area (mm^3)
Actuator	52	34.3	Elongation: 2.25	922
			Contraction: 0.461	
			Lateral:0.074	

Ring & Segment	Radius r (mm)	Circumscribed radius r_c (mm)	Inscribed radius r_i (mm)	Connector (mm)	
				Width a	Thickness h
Bigger prototype	65	75	35	49	25
Smaller Prototype	42	52	25.5	49	33

**Robot**		Length (mm)	Width (mm)	Height (mm)	Weight (g)	Foot diameter of suction cup (mm)
Segment	Bigger prototype	122	133	133	324	26
	Smaller prototype	90	100	101	157	
SoCL robot		300	133	133	660	

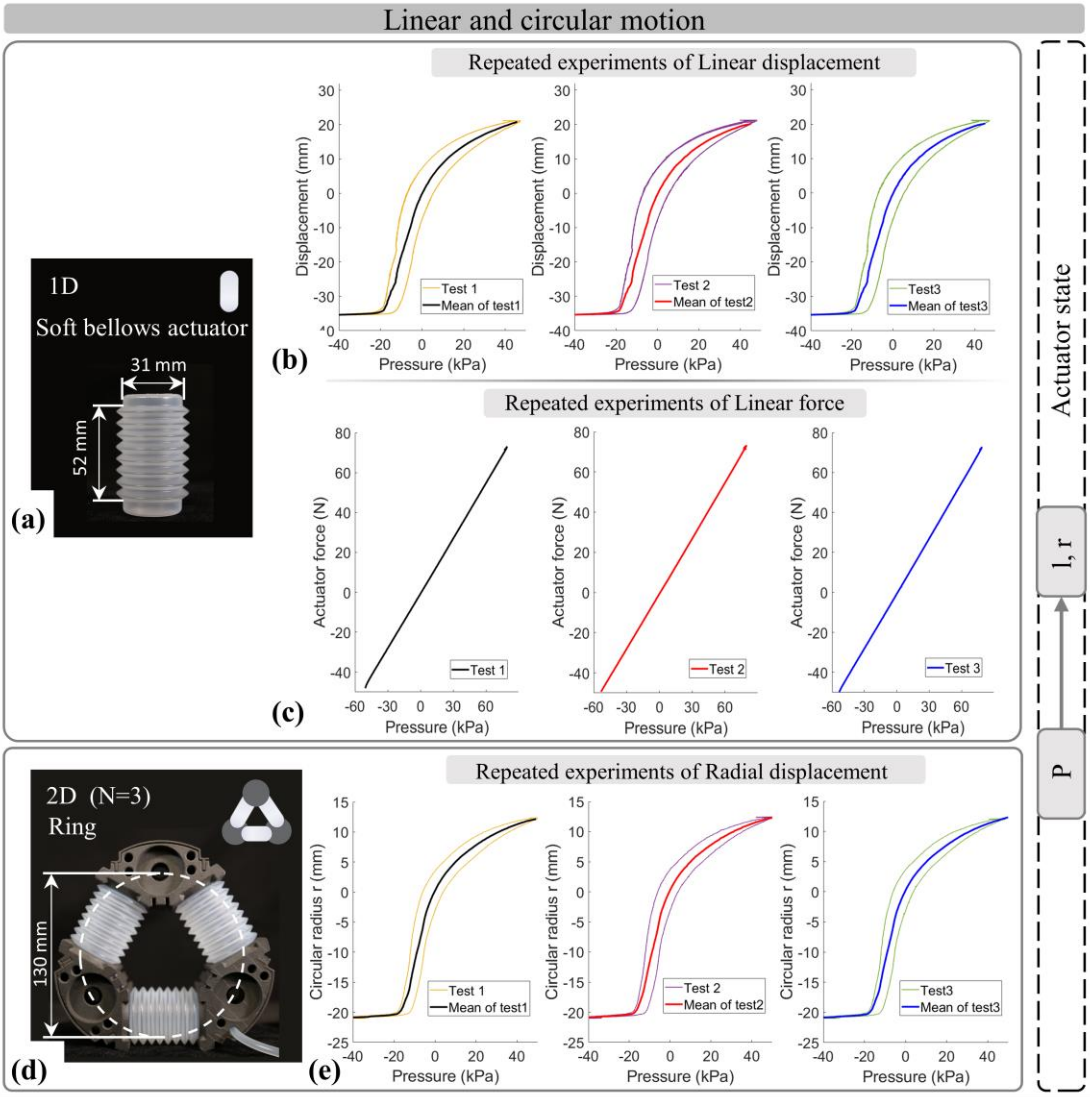

Supplementary Fig. S8. The respective data of repeated experiments, covering the relationship between (b) linear displacement and (c) linear force of (a) the soft bellows actuator to the changed pressure, and the relationship between (e) radial displacement of (d) the ring to the changed pressure. The shown curves involve the experimental data and the mean of it.

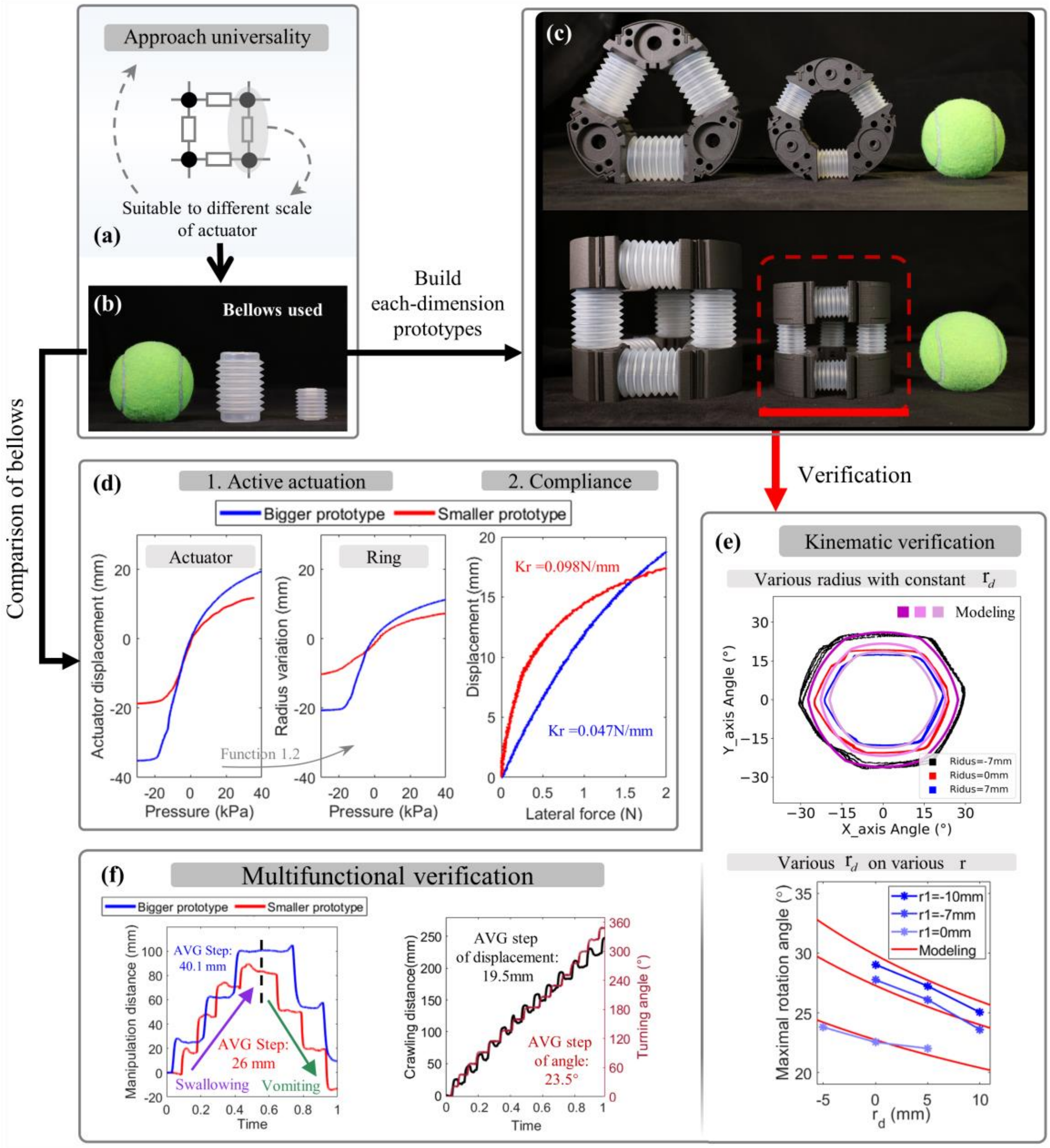

Supplementary Fig. S9. The approach's universality to actuator, and small prototypes with performance testing and verification of model. (a) With the universality to actuator, this cross-linked actuator network allows diverse bellow used. Therefore, we constructed the small prototypes (c) used the small bellows (b), compared with the prototypes of normal size. This exhibited the similar capability of linear/radial motion (d), bidirectional swallowing and locomotion functions (f), while has the smaller average step of operation. The performance of coupled motion verified the universality of the proposed kinematic model (e), with the matching curve with the estimated results.
